# Apocynin prevents mitochondrial burdens, microglial activation, and pro-apoptosis induced by a toxic dose of methamphetamine in the striatum of mice via inhibition of p47phox activation by ERK

**DOI:** 10.1186/s12974-016-0478-x

**Published:** 2016-01-18

**Authors:** Duy-Khanh Dang, Eun-Joo Shin, Yunsung Nam, Sungwoo Ryoo, Ji Hoon Jeong, Choon-Gon Jang, Toshitaka Nabeshima, Jau-Shyong Hong, Hyoung-Chun Kim

**Affiliations:** Neuropsychopharmacology and Toxicology Program, College of Pharmacy, Kangwon National University, Chunchon, South Korea; Department of Biological Sciences, College of Natural Sciences, Kangwon National University, Chunchon, South Korea; Department of Pharmacology, College of Medicine, Chung-Ang University, Seoul, South Korea; Department of Pharmacology, School of Pharmacy, Sungkyunkwan University, Suwon, South Korea; Department of Regional Pharmaceutical Care and Sciences, Graduate School of Pharmaceutical Sciences, Meijo University, Nagoya, Japan; NPO, Japanese Drug Organization of Appropriate Use and Research, Nagoya, Japan; Neuropharmacology Section, Laboratory of Toxicology and Pharmacology, National Institute of Environmental Health Sciences, Research Triangle Park, Durham, NC USA

**Keywords:** Methamphetamine toxicity, Phosphorylation of ERK1/2, p47phox knockout mice, Mitochondria, Cleaved PKCδ, Oxidative stress, Microglia of M1 phenotype, Pro-apoptosis, Striatum, Dopamine

## Abstract

**Background:**

Activation of NADPH oxidase (PHOX) plays a critical role in mediating dopaminergic neuroinflammation. In the present study, we investigated the role of PHOX in methamphetamine (MA)-induced neurotoxic and inflammatory changes in mice.

**Methods:**

We examined changes in mitogen-activated protein kinases (MAPKs), mitochondrial function [i.e., mitochondrial membrane potential, intramitochondrial Ca^2+^ accumulation, mitochondrial oxidative burdens, mitochondrial superoxide dismutase expression, and mitochondrial translocation of the cleaved form of protein kinase C delta type (cleaved PKCδ)], microglial activity, and pro-apoptotic changes [i.e., cytosolic cytochrome c release, cleaved caspase 3, and terminal deoxynucleotidyl transferase dUDP nick-end labeling (TUNEL) positive populations] after a neurotoxic dose of MA in the striatum of mice to achieve a better understanding of the effects of apocynin, a non-specific PHOX inhibitor, or genetic inhibition of p47phox (by using p47phox knockout mice or p47phox antisense oligonucleotide) against MA-induced dopaminergic neurotoxicity.

**Results:**

Phosphorylation of extracellular signal-regulated kinases (ERK1/2) was most pronounced out of MAPKs after MA. We observed MA-induced phosphorylation and membrane translocation of p47phox in the striatum of mice. The activation of p47phox promoted mitochondrial stresses followed by microglial activation into the M1 phenotype, and pro-apoptotic changes, and led to dopaminergic impairments. ERK activated these signaling pathways. Apocynin or genetic inhibition of p47phox significantly protected these signaling processes induced by MA. ERK inhibitor U0126 did not exhibit any additional positive effects against protective activity mediated by apocynin or p47phox genetic inhibition, suggesting that ERK regulates p47phox activation, and ERK constitutes the crucial target for apocynin-mediated inhibition of PHOX activation.

**Conclusions:**

Our results indicate that the neuroprotective mechanism of apocynin against MA insult is via preventing mitochondrial burdens, microglial activation, and pro-apoptotic signaling process by the ERK-dependent activation of p47phox.

**Electronic supplementary material:**

The online version of this article (doi:10.1186/s12974-016-0478-x) contains supplementary material, which is available to authorized users.

## Background

It has been well-recognized that high doses of methamphetamine (MA) result in impaired nigrostriatal dopaminergic systems in both rodents [1–4] and primates [5]. Although the pathogenesis on the MA-induced dopaminergic neurotoxicity remains to be further elucidated, this neurotoxicity may be, at least in part, related to oxidative stress [3, 4, 6–10], inflammatory changes [4, 6, 11, 12], and pro-apoptosis [4, 9, 10, 13–16]. Thus, dopaminergic neurotoxicity induced by high doses of MA may be a possible Parkinson’s disease (PD) model [17–21].

Furthermore, previous investigations have suggested that humans who abuse MA have an increased risk for PD later in life [22–25]. Earlier postmortem studies reported reductions in dopamine levels, tyrosine hydroxylase (TH) expression, and dopamine transporter (DAT) binding in the striatum of MA abusers [26], and these changes paralleled neurochemical changes in Parkinson’s disease (PD) patients [27, 28].

Accumulated evidence indicates that MA can also cause oxidative stress by shifting the balance between reactive oxygen species (ROS) production and the capacity of antioxidant systems to scavenge ROS [3, 4, 29–31]. Recently, we have proposed that MA-induced mitochondrial oxidative stress and mitochondrial dysfunction promotes dopaminergic degeneration [4, 8]. Interestingly, NADPH oxidase (PHOX) activation was observed in response to mitochondrial ROS formation in human leukocytes [32].

PHOX is a multiunit enzyme that catalyzes the reduction of molecular oxygen to form superoxide radicals and is composed of gp91phox, p22phox, p47phox, p67phox, p40phox, and small GTPase Rac (Rac1 or Rac2) subunits. Under basal conditions, p47phox, p67phox, and p40phox are present in the cytosol as a complex [33], and Rac is bound to its inhibitory protein, RhoGDP-dissociation inhibitor (RhoGDI) [34]. These subunits are separated from the transmembrane gp91phox and p22phox subunits [33, 34]. Upon activation, the p47phox subunit gets phosphorylated and translocates to the membrane as a complex to assemble with gp91phox, p22phox, and membrane-translocated Rac to form an active PHOX capable of reducing oxygen to a superoxide radical to generate microglial [35–38] and/or mitochondrial-derived ROS [32] and possibly neuronal and astroglial ROS [35, 39].

Microglia-mediated neuroinflammation has been linked to multiple neurodegenerative diseases, including PD [35, 37–45]. One recent therapeutic strategy has been to deviate from conventional anti-inflammatory targets and inhibit upstream mediators, such as PHOX [35]. Once activated, PHOX produces extracellular and intracellular reactive oxygen species, which are critical in initiating and maintaining neuroinflammatory responses, leading to progressive dopaminergic neurodegeneration [42, 43, 46]. For example, activated microglia secrete a variety of toxic factors, such as tumor necrosis factor α, interleukin-1, and other pro-inflammatory cytokines, which work in concert to cause neuronal damage [41]. Hong and colleagues have recognized PHOX as a key mediator in bridging neuroinflammation and progressive dopaminergic neurodegeneration [42, 43, 47].

Importantly, a recent investigation demonstrated that treatment with apocynin, a non-specific inhibitor of PHOX [48], results in a significant reduction in MA-induced dopamine-release from rat striatal slices [49]. Furthermore, Park et al. [50] found that MA (10 μM) induces an increase in phosphorylation of the p47phox subunit and subsequently enhanced PHOX activity in endothelial cells. However, the information of PHOX in the MA-induced neurotoxicity in vivo remains unknown. Thus, we investigated whether apocynin affects dopaminergic neurotoxicity induced by MA in mice, and whether apocynin modulates p47phox in our system, because p47phox acts as a connector between the components of the membrane and the cytoplasm [36, 51, 52]. We suggested here for the first time that inhibition of the extracellular signal-regulated kinase (ERK)-dependent phosphorylation and membrane translocation of p47phox are critical for apocynin-mediated protective potentials against oxidative stress (mitochondria > cytosol), neuroinflammatory change, and pro-apoptotic pathway induced by MA and that these morbid events require pro-apoptotic scenarios induced by a toxic dose of MA.

## Methods

### Animals

All animals were treated in accordance with the National Institutes of Health (NIH) Guide for the Humane Care and Use of Laboratory Animals (NIH Publication No. 85-23, 1985; www.dels.nas.edu/ila). The present study was performed in accordance with the Institute for Laboratory Research (ILAR) Guidelines for the Care and Use of Laboratory Animals, and the animal experimental procedure was approved by the Institutional Animal Care and Use Committee (IACUC) of Kangwon National University (#KIACUC-12-0016). Mice were maintained under a 12-h light:12-h dark cycle and fed ad libitum. They were adapted to these conditions for 2 weeks prior to the experiment. Wild-type C57BL/6 and p47phox knockout mice were purchased from Jackson Laboratories (Bar Harbor, ME, USA) [53].

To evaluate the effect of apocynin or p47phox antisense oligonucleotide on the MA-induced pro-apoptosis, we employed 10-week-old male ICR mice (Taconic Farms, Inc., Samtako Bio Korea, O-San, South Korea) because our previous reports [4, 8, 9] indicated that the C57BL/6 background does not exhibit MA-induced terminal deoxynucleotidyl transferase dUDP nick-end labeling (TUNEL)-positive cells in the striatum, but does so in Taconic ICR mice.

### Drug treatment

Although the 4 × 7–10 mg/kg paradigm of MA administration is currently the most frequently used model that mimics an acute toxic dose of MA [6], we selected a toxic dose (35 mg/kg, i.p.) of MA in the present study because this paradigm is more sensitive than the 4 × 7 mg/kg paradigm for producing more significant dopaminergic protective effects by apocynin or p47phox gene knockout (Additional file 1; Additional file 2: Figs. S1–4).

Mice were treated with a single dose of MA (35 mg/kg, i.p.) or saline and sacrificed 30 min, 1 h, 2 h, 4 h, 6 h, and 1 day after MA treatment (Additional file 2: Fig S5) to examine phosphorylation and membrane translocation of p47; mitochondrial translocation of cleaved cleaved form of protein kinase C delta type (PKCδ); mitochondrial membrane potential; intramitochondrial Ca^2+^ level; ROS formation; phosphorylations of ERK, p38 mitogen-activated protein kinase (p38), and c-Jun N-terminal kinase (JNK); and changes in TH-, ionized calcium binding adaptor molecule 1 (Iba-1)-, and mitochondrial manganese-dependent superoxide dismutase (MnSOD) expression and in dopamine and its metabolite levels. Apocynin (Sigma-Aldrich, St. Louis, MO, USA) was dissolved in dimethyl sulfoxide (DMSO) and then diluted in sterile saline immediately prior to use at a concentration of 50 mg/ml. The final DMSO concentration was 10 % (*v*/*v*). Administration of apocynin (50 mg/kg, i.p.) was conducted once daily for seven consecutive days. The last dose of apocynin was given 30 min before MA injection (Additional file 2: Fig S5). The dose of apocynin was determined based on a previous study [49]. U0126 (ERK inhibitor; Tocris Bioscience, Ellisville, MO, USA) was dissolved in DMSO as a stock solution and then stored at −20 °C. U0126 was diluted in sterile saline immediately before use at a concentration of 2 μg/μL. The final DMSO concentration was 10 % (*v*/*v*). U0126 (2 μg, i.c.v.) was given 1 h before MA injection (Additional file 2: Fig. S5). The dose of U0126 was determined based on a previous study [54].

### Guide cannula building and intracerebroventricular infusion with p47phox sense oligonucleotide (p47phox SO) or p47phox antisense oligonucleotide (p47phox ASO)

A stainless steel guide cannula (AG-4; Eicom, Kyoto, Japan) was implanted into the right lateral ventricle (stereotaxic coordinates: 0.5 mm posterior to bregma, 1 mm right to the midline, and 2 mm ventral to the dura, according to the atlas of Franklin and Paxinos) as described previously [4, 8, 55]. One day after guide cannula implantation, Taconic ICR mice received a single injection of MA (35 mg/kg, i.p.). p47phox antisense oligonucleotide (p47phox ASO; 5′-GGTGTCCCCCATGGCTGGGCCG) or control p47phox sense oligonucleotide (p47phox SO; 5′-CGGCCCAGCCATGGGGGACACC) [GenBank accession number: AB002663.1] was microinfused into the lateral ventricle at a dose of 2.5 μg/μL at 4 and 0.5 h before, and at 4 h after MA injection. P47phox SO and p47phox ASO used here were phosphorothioated on the two terminal bases of the 5′-end and three terminal bases of the 3′-end (Bioneer Corporation, Daejeon, South Korea). Microinfusion into the lateral ventricle was performed through a microinfusion cannula (AMI-4, Eicom) at a rate of 1 μL/min using a microinjection pump (CMA/100, CMA, Solna, Sweden). The microinfusion cannula was kept in place for 1 min after infusion to avoid backflow.

### Preparation of cytosolic and membrane fractions for Western blot analysis

Cytosolic and membrane fractions were prepared as described previously with minor modifications [56]. Animal tissues were collected and homogenized in ice-cold lysis buffer (pH 7.4) containing 25 mmol/L Tris, 250 mmol/L NaCl, 3 mmol/L ethylenediaminetetraacetic acid (EDTA), and protease inhibitor cocktail (Sigma-Aldrich, St. Louis, MO, USA) using Dounce homogenizer. The lysates were loaded onto sucrose in lysis buffer and centrifuged at 1600×*g* for 15 min; the supernatant above the sucrose gradient was utilized as the cytosolic fraction after centrifugation at 150,000×*g* for 30 min at 4 °C. The resulting pellets were resuspended with lysis buffer containing 1 % Triton X-100 and used as the membrane fraction.

### Preparation of cytosolic and mitochondrial fraction for Western blot and neurochemical analyses

Preparation of cytosolic and mitochondrial fraction was performed as described previously [4, 8]. Briefly, striatal tissues were collected and homogenized in ice-cold homogenization buffer containing 0.25 M sucrose, 0.5 mM potassium ethylene glycol-bis(2-aminoethyl ether)-N,N,N′,N′-tetraacetic acid (EGTA), 10 mM Tris–HCl (pH 7.4), and protease inhibitor cocktail (Sigma-Aldrich, St. Louis, MO, USA) using Dounce homogenizer. Homogenates were centrifuged at 2000×*g* for 10 min to remove nuclei and unbroken cells. Supernatants were then centrifuged at 12,000×*g* for 15 min to obtain crude mitochondrial pellets and cytosolic supernatant. Crude mitochondrial pellets were suspended in 3 % Ficoll 400 (Sigma-Aldrich) in Ficoll dilution buffer containing 0.25 M mannitol, 60 mM sucrose, 0.1 mM potassium EGTA, and 10 mM Tris–HCl (pH 7.4). A Ficoll density gradient was constructed by pouring crude mitochondrial suspension in 3 % Ficoll over 6 % Ficoll 400 solution. Purified mitochondrial pellets, which were obtained by centrifugation at 11,500×*g* for 10 min, were resuspended in buffer containing 210 mM mannitol, 70 mM sucrose, 5 mM 4-(2-hydroxyethyl)-1-piperazineethanesulfonic acid (HEPES), and protease cocktail (pH 7.4). For Western blot, mitochondrial pellets were lysed in 100 μL of lysis buffer.

### Western blot analysis

For Western blot analysis of phospho (p)-p47phox, p-ERK, p-p38, p-JNK, Iba-1, cleaved caspase 3, and TH, striatal tissues were lysed in buffer containing a 200 mM Tris–HCl (pH 6.8), 1 % SDS, 5 mM EGTA, 5 mM EDTA, 10 % glycerol, 1 × phosphatase inhibitor cocktail I (Sigma-Aldrich, St. Louis, MO, USA), and 1 × protease inhibitor cocktail (Sigma-Aldrich, St. Louis, MO, USA). Lysate was centrifuged at 12,000×*g* for 30 min, and the supernatant fraction was used for Western blot analysis as described previously [4, 9]. Proteins (20 μg/lane) were separated by 8 % or 10 % sodium dodecyl sulfate polyacrylamide gel electrophoresis (PAGE) and transferred onto the polyvinylidene fluoride (PVDF) membranes. Following transfer, the membranes were preincubated with 5 % non-fat milk for 30 min and incubated overnight at 4 °C with primary antibody against p47phox [1:500; Chemicon (EMD Millipore), Temecula, MA, USA], p-p47phox at Ser345 (1:1000; Sigma-Aldrich, St. Louis, MO, USA), Na^+^/K^+^-ATPase α1 subunit (1:1000; Abcam, Cambridge, UK), ERK (1:5000; Cell Signaling Technology, Danvers, MA, USA), p-ERK (1:1000; Cell Signaling Technology), p38 (1:2000; Cell Signaling Technology), p-p38 (1:1000; Cell Signaling Technology), JNK (1:5000; Cell Signaling Technology), p-JNK (1:1000; Cell Signaling Technology), cleaved PKCδ (1:2000; Santa Cruz Biotechnology), cytochrome c (1:500; Santa Cruz Biotechnology), MnSOD (1:10000; kindly gifted by Dr. Kanefusa Kato at Aichi Prefectural Colony, Kasugai, Japan) [18], cleaved caspase 3 (1:1000; Cell Signaling Technology), Iba-1 (1:500, Abcam), TH [1:5000; Chemicon (EMD Millipore)], β-actin (1:50000; Sigma-Aldrich), or COX IV (1:10000; Cell Signaling Technology). Membranes were then incubated with HRP-conjugated secondary anti-rabbit IgG (1:1000, GE healthcare, Piscataway, NJ, USA), anti-mouse IgG (1:1000, Sigma-Aldrich), or anti-goat IgG (1:1000, Sigma-Aldrich) for 2 h. Subsequent visualization was performed using an enhanced chemiluminescence system (ECL plus®, GE Healthcare, Arlington Heights, IL, USA). Relative intensities of the bands were quantified by PhotoCapt MW (version 10.01 for Windows; Vilber Lourmat, Marne la Vallée, France) and then normalized to the intensity of β-actin (whole lysate or cytosolic fraction), COX IV (mitochondrial fraction), or Na^+^/K^+^-ATPase α1 subunit (membrane fraction) [4].

### Mitochondrial preparation for in vivo measurement of mitochondrial membrane potential and intramitochondrial Ca^2+^ level

Mitochondria were isolated as described previously with minor modifications [4, 8]. The animals were anesthetized with sodium pentobarbital (60 mg/kg) and perfused transcardially with 30 mL ice-cold homogenization buffer (250 mM sucrose, 20 mM HEPES, 1 mM EDTA, pH 7.2). The animals were then decapitated, and the striatum was dissected out, rinsed in 9 mL homogenization buffer, and processed using a tissue homogenizer. All subsequent steps were conducted at 4 °C. The resulting homogenate was centrifuged (10 min, 1300×*g*). The supernatant was removed and centrifuged again (10 min, 10,000×*g*), and the pellet was gently resuspended (four strokes) in 30 mL homogenization buffer using a hand-held homogenizer and centrifuged (10 min, 10,000×*g*). The resulting pellet was resuspended and rinsed in EDTA-free homogenization buffer. Then the mitochondrial pellet was resuspended in 250 mM sucrose to a final concentration of ~20 mg/mL and placed on ice. The entire mitochondrial preparation took <1 h to complete.

### Mitochondrial membrane potential

Mitochondrial membrane potential (MMP) was measured as described previously [4] using 5,5′,6,6′-tetrachloro-1,1′,3,3′-tetraethylbenzimidazolycarbocyanine iodide dye (JC-1; Molecular Probes), which exists as a green fluorescent monomer at low membrane potential, but reversibly forms red fluorescent “J-aggregates” at polarized mitochondrial potentials. Briefly, 250-μg aliquots of isolated mitochondrial protein were suspended in respiration buffer [250 mM sucrose, 20 mM HEPES, 2 mM MgCl_2_, 2.5 mM inorganic phosphates (pH 7.2), and 10 mM succinate (5 mM glutamate and 2.5 mM maleate produced similar results in all paradigms)] in a final volume of 200 μL. The energized mitochondria were then incubated at 37 °C in the presence of 10 μM JC-1 for 30 min, after which fluorescence was measured with a fluorescent plate reader (Molecular Devices). The relative amount of mitochondrial polarization was quantified by taking the ratio of emission from 590 to 535 nm, respectively, with excitation at 490 nm.

### Intramitochondrial Ca^2+^ levels

Intramitochondrial Ca^2+^ levels were measured as described previously [4]. Mitochondrial fractions (250 μg) from striatal tissues were incubated in the presence of Rhod-2-AM (5 μM) for 60 min at 37 °C and washed three times with Ca^2+^-free Locke’s solution. This reduced form of Rhod-2-AM is a colorless, nonfluorescent dye that has a net positive charge, which promotes sequestration into mitochondria. Then, the dye is oxidized in the mitochondria where the AM ester is cleaved, trapping the dye in the mitochondria. Fluorescence was quantified with a fluorescent plate reader (Molecular Devices), with excitation and emission wavelengths of 549 and 581 nm, respectively.

### Determination of ROS formation

The ROS formation in the striatum was assessed by measuring the conversion from 2′,7′-dichlorofluorescin diacetate (DCFH-DA) to dichlorofluorescin (DCF) [8]. Cytosolic or mitochondrial fraction was added to a tube containing 2 mL of PBS with 10 nmol of DCFH-DA, dissolved in methanol. Mixture was incubated at 37 °C for 3 h, and then fluorescence was measured at a 480-nm excitation and 525-nm emission. DCF is used as a standard.

### Immunocytochemistry

Immunocytochemistry was performed as described previously [4]. Mice were perfused transcardially with 50 mL of ice-cold PBS (10 mL/10 g body weight) followed by 4 % paraformaldehyde (20 mL/10 g body weight). Brains were removed and stored in 4 % paraformaldehyde overnight. Series of every sixth sections (35 μm thickness, 210 μm apart) from striatum were selected and subjected to immunocytochemistry. Sections were blocked with PBS containing 0.3 % hydrogen peroxide for 30 min and then incubated in PBS containing 0.4 % Triton X-100 and 1 % normal serum for 20 min. After a 48-h incubation with primary antibody against TH [1:500; Chemicon (EMD Millipore)] and Iba-1 (1:500, Wako Pure Chemical Industries, Chuo-ku, Osaka, Japan), sections were incubated with the biotinylated secondary antibody (1:1000; Vector Laboratories, Burlingame, CA, USA) for 1 h. The sections were then immersed in a solution containing avidin–biotin peroxidase complex (Vector Laboratories) for 1 h, and 3,3′-diaminobenzidine was utilized as the chromogen.

To examine TH-immunoreactivity, digital images were acquired at ×4 objective magnification using an Olympus microscope (BX51; Olympus) and a digital microscope camera (DP72; Olympus). ImageJ version 1.47 software (National Institutes of Health, Bethesda, MD, USA) was employed to measure the TH-immunoreactivity as described previously [21]. Briefly, the entire striatal region from each section was selected as the region of interest (ROI). Threshold values for hue (0–100), saturation (0–255), and brightness (175–255) were set in the “Adjust Color Threshold” dialog box, and then the mean density was measured.

### Morphological changes in microglia

To analyze morphological changes in microglia, digital images of Iba-1-immunostained sections were obtained at ×40 objective magnification under an upright microscope (BX51; Olympus) using an attached digital microscope camera (DP72; Olympus). Each section was acquired in 11 planes of focusing, and then the images were stacked and integrated into one image for morphological analysis [57]. The resolution of the resulting images was 1360 pixels × 1024 pixels (350 μm × 263 μm).

Skeleton analysis was performed as described previously [58–60]. For skeleton analysis, the resulting images were subjected to background subtraction, converted to 8 bit, and binarized using ImageJ version 1.47 software. The “Skeletonize3D” plugin (fiji.sc/Skeletonize3D) and “AnalyzeSkeleton” plugin (fiji.sc/AnalyzeSkeleton) were applied to skeletonize and analyze skeleton, as shown in Additional file 2: Fig. S8a. The number of branches, the number of junctions, the number of triple points (junctions with exactly three branches), the average branch length, and summed branch length were determined.

The cell size and cell body size in the area were determined as described previously [61, 62], using ImageJ version 1.47 software. Background was subtracted from each image to correct uneven background. To measure the cell size, all pixels that were darker than the background were selected by the auto-threshold command. Cell bodies were selected by manual intensity selection, as shown in Additional file 2: Fig. S8c. The “Analyze Particles” command was employed to measure the cell size and cell body size. The number of cell bodies was counted to normalize the cell size and cell body size per cell. The cell body size to cell size ratio (%) was also determined.

### Reverse transcription and polymerase chain reaction (RT-PCR)

Total RNA was isolated from striatal tissues using an RNeasy Mini Kit (Qiagen, Valencia, CA, USA) according to the manufacturer’s instructions. Reverse transcription reactions were carried out using the RNA to cDNA EcoDry Premix (Clontech, Palo Alto, CA, USA) with a 1-h incubation at 42 °C. PCR amplification was performed for 35 cycles of denaturation at 94 °C for 1 min, annealing at 60 °C for 2 min, and extension at 72 °C for 1 min. Primer sequences [21] for PCR amplification are listed in Table 1. PCR products were separated on 2 % agarose gels containing ethidium bromide and visualized under ultraviolet light. Quantitative analysis of RNA was performed using PhotoCapt MW (version 10.01 for Windows; Vilber Lourmat).

**Table 1:** Gene primer sequences for RT-PCR analysis

Gene	Forward primer (5′-3′)	Reverse primer (5′-3′)
Arginase 1	GAACACGGCAGTGGCTTTAAC	TGCTTAGCTCTGTCTGCTTTGC
CD206	TCTTTGCCTTTCCCAGTCTCC	TGACACCCAGCGGAATTTC
CD16	TTTGGACACCCAGATGTTTCAG	GTCTTCCTTGAGCACCTGGATC
CD32	AATCCTGCCGTTCCTACTGATC	GTGTCACCGTGTCTTCCTTGAG
CD86	TTGTGTGTGTTCTGGAAACGGAG	AACTTAGAGGCTGTGTTGCTGGG
GAPDH	ACCACAGTCCATGCCATCAC	TCCACCACCCTGTTGCTGTA

### TUNEL staining

For TUNEL staining, a series of every sixth section (35 μm thickness, 210 μm apart) from striatum was selected. TUNEL staining was performed using the FragEL DNA fragmentation detection kit (QIA33; Calbiochem, La Jolla, CA, USA) according to the manufacturer’s protocol [4, 8, 9]. Briefly, sections were permeabilized by incubation with 20 mg/ml proteinase K, and then incubated with 3 % hydrogen peroxide to block endogenous peroxidase activity. After immersion in the terminal deoxynucleotidyl transferase (TdT) equilibration buffer, sections were incubated with biotinylated deoxynucleotides and TdT enzyme. Sections were then immersed in streptavidin-peroxidase complex with diaminobenzidine tetrahydrochloride as the chromogen. Counterstaining was performed using methyl green, which was provided in the kit. Digital images from each quadrant of the striatum (dorsal-medial, dorsal-lateral, ventral-media, ventral-lateral) were acquired [15, 16] at ×40 objective magnification using an Olympus microscope (BX51; Olympus) and a digital microscope camera (DP72; Olympus). Cell counting was performed blindly. Apoptotic cells were identified based on the rounded, shrunken nature of the cytoplasm and nucleus and on the intense staining of the nucleus. After counting, a mean value was obtained by averaging the counts of each quadrant from five sections for each animal [45].

### Measurements of dopamine, 3,4-dihydroxyphenylacetic acid, and homovanillic acid

Mice were sacrificed by cervical dislocation, and the brains were removed. The striatum was dissected, immediately frozen on dry ice, and stored at −70 °C before assays were performed. Tissues were weighed, ultrasonicated in 10 % perchloric acid, and centrifuged at 20,000×*g* for 10 min. The levels of dopamine (DA) and its metabolites 3,4-dihydroxyphenylacetic acid (DOPAC) and homovanillic acid (HVA) were determined by HPLC coupled with an electrochemical detector, as described previously [4, 21]. Supernatant aliquots (20 μL) were injected into an HPLC equipped with a C18 column with 3 μm particle size (Waters). The mobile phase was comprised of 26 mL of acetonitrile, 21 mL of tetrahydrofuran, and 960 mL of 0.15 M monochloroacetic acid (pH 3.0) containing 50 mg/L of EDTA and 200 mg/mL of sodium octyl sulfate. The amount of DA was determined by comparison of peak areas of tissue samples with standard, and was expressed in nanograms per gram of wet tissue.

### Statistical analyses

Data were analyzed using IBM SPSS ver. 21.0 (IBM, Chicago, IL, USA). One-way analysis of variance (ANOVA) (time) or three-way ANOVA (MA × p47phox inhibition × ERK inhibition) was employed for the statistical analyses. Post hoc Fisher’s least significant difference pairwise comparison tests were then conducted. *P* values <0.05 were considered to be significant.

## Results

### Methamphetamine treatment significantly promoted phosphorylation and membrane translocation of p47phox and phosphorylation of mitogen-activated protein kinase (MAPK) in the striatum of wild-type mice

**Fig. 1:**
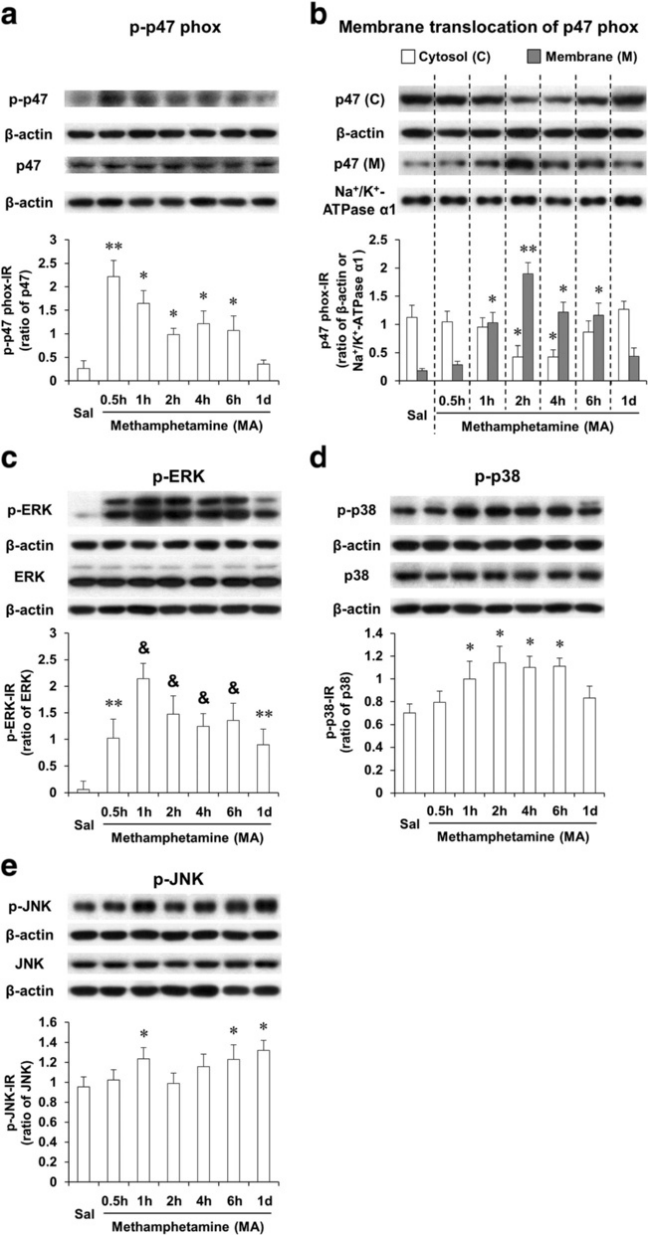
MA-induced activation of p47phox and MAPKs. Phosphorylation (**a**) and membrane translocation (**b**) of p47phox and phosphorylations of ERK (**c**), p38 (**d**), and JNK (**e**) after MA treatment (35 mg/kg, i.p.). *Sal* saline. Each value is the mean ± S.E.M. of six animals. ^*^ *P* < 0.05, ^**^ *P* < 0.01, and ^&^ *P* < 0.001 vs. saline (one-way ANOVA was followed by Fisher’s LSD pairwise comparisons)

It is recognized that phosphorylation of p47phox constitutes one of the key intracellular events associated with PHOX activation, and Ser345 phosphorylation of p47phox by the MAPK protein plays a critical role in the potentiation of PHOX activation by pro-inflammatory agents [36, 51, 52]. Thus, we examined the levels of p47phox phosphorylation and membrane translocation induced by MA. In addition, we investigated MA-induced phosphorylations of ERK1/2, p38, and JNK. As shown in Fig. 1, we examined phosphorylation (Fig. 1a) and translocation (Fig. 1b) of p47phox 30 min, 1 h, 2 h, 4 h, 6 h, and 1 day after MA treatment in wild-type (WT) mice. MA-induced phosphorylation of p47phox was most pronounced at 30 min (Fig. 1a), while translocation of p47phox was most evident at 2 h (Fig. 1b).

Treatment with MA resulted in strong inductions in p-ERK1/2 in WT mice. MA-induced initial increase in p-ERK1/2 was observed 30 min (*P* < 0.01) later. The most significant increase in p-ERK1/2 was noted 1 h (*P* < 0.001) after MA, and p-ERK1/2 expression remained significantly elevated (*P* < 0.01) 1 day later (Fig. 1c). Although MA-induced increases in p-p38 (Fig. 1d) and p-JNK (Fig. 1e) were observed in WT mice, these increases were much less pronounced than that in p-ERK1/2 (Fig. 1d, e).

### ERK1/2 activation is required for methamphetamine-induced p47phox phosphorylation and translocation; ERK1/2 inhibitor U0126, apocynin, or p47phox knockout attenuates MA-induced activation of ERK1/2 and p47phox

**Fig. 2:**
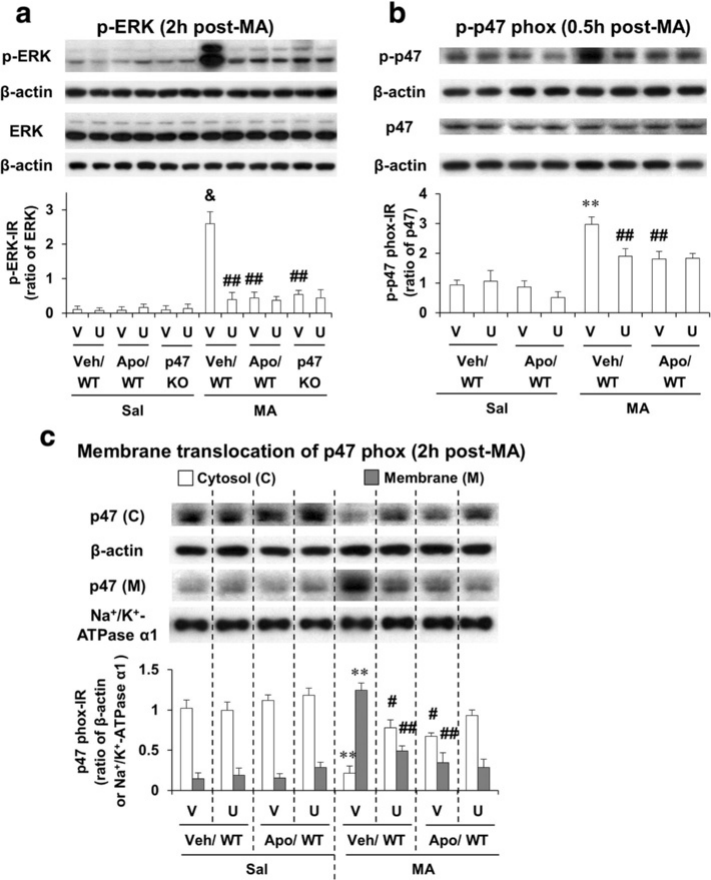
Effects of U0126 and apocynin or p47phox knockout against MA-induced activations in ERK and p47phox. Phosphorylation of ERK (**a**) and phosphorylation (**b**) and membrane translocation (**c**) of p47phox after MA (35 mg/kg, i.p.). *WT* wild-type mice, *p47 KO* p47phox knockout mice, *Sal* saline, *U* U0126 (2 μg, i.c.v.), *Apo* apocynin (50 mg/kg, i.p.), *V or Veh* vehicle [10 % (*v*/*v*) DMSO] for U0126 or apocynin. Each value is the mean ± SEM of six animals. ^**^ *P* < 0.01, ^&^ *P* < 0.001 vs. vehicle/WT with saline. ^#^ *P* < 0.05 or ^##^ *P* < 0.01 vs. vehicle/WT with MA (three-way ANOVA was followed by Fisher’s LSD pairwise comparisons)

As shown in Fig. 2, p-ERK1/2 expression was not altered significantly without MA in WT and p47phox knockout (KO) mice. MA-induced phosphorylation of ERK1/2 was most prominent 1 h later, and thus, we focused on this time. The specific ERK inhibitor U0126 or apocynin significantly attenuated (*P* < 0.01) MA-induced increase in p-ERK1/2 expression in WT mice. Consistently, MA-induced significant increase in p-ERK1/2 in WT mice was not observed in p47phox KO mice. However, U0126 treatment did not exhibit any additional effects in response to attenuation mediated by apocynin or p47phox KO (Fig. 2a).

We then examined the effect of U0126 or apocynin against phosphorylation of p47phox 30 min post-MA. Either U0126 or apocynin significantly attenuated (*P* < 0.01) phosphorylation of p47phox in WT mice. U0126 treatment did not alter inhibited phosphorylation of p47phox by apocynin (Fig. 2b). Since membrane translocation of p47phox was maximally induced 2 h post-MA, we examined the effect of U0126 or apocynin against membrane translocation of p47phox at that time. Either U0126 or apocynin significantly inhibited (*P* < 0.01) p47phox membrane translocation in WT mice. U0126 treatment failed to affect membrane translocation of p47phox mediated by apocynin (Fig. 2c).

### ERK inhibitor U0126, apocynin, or genetic depletion of p47phox protects MA-induced mitochondrial dysfunction; U0126 does not significantly affect the protection mediated by apocynin or p47phox knockout

We have demonstrated that multiple doses of MA (i.e., four injections of 7 mg/kg MA, intraperitoneally at 2 h intervals) impair MMP and intramitochondrial Ca^2+^ accumulation in the striatum of mice [4, 8]. We examined here whether a toxic dose of MA affects MMP and intramitochondrial Ca^2+^ level and whether inhibition of ERK, PHOX, or p47phox gene modulates these mitochondrial parameters in the striatum of mice.

**Fig. 3:**
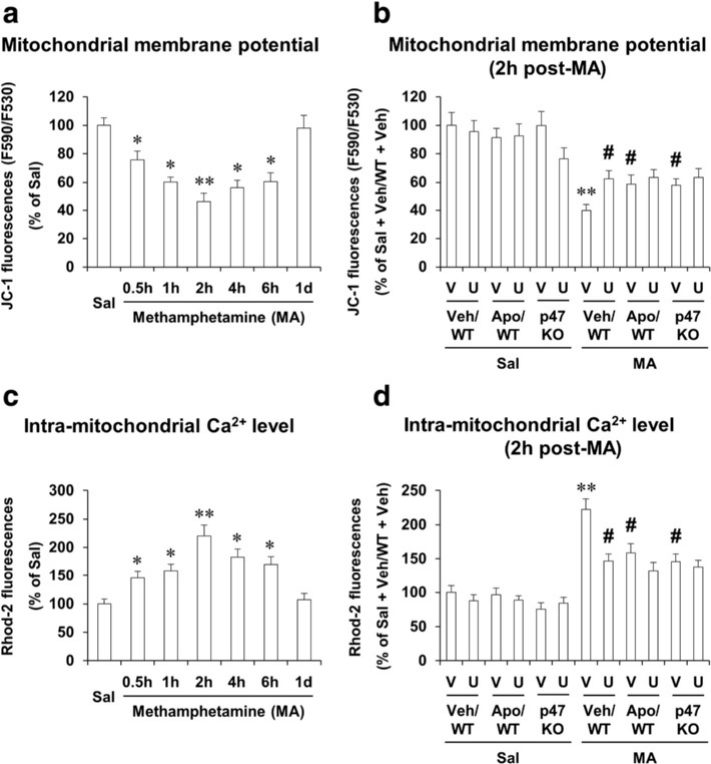
Effects of U0126, apocynin, or p47phox knockout on mitochondrial dysfunction after MA treatment. MA-induced changes in mitochondrial membrane potential (**a**) and intramitochondrial Ca^2+^ level (**c**), and effects of ERK1/2 inhibitor U0126, apocynin, or p47phox gene knockout on mitochondrial membrane potential (**b**) and intramitochondrial Ca^2+^ level (**d**). *WT* wild-type mice, *p47 KO* p47phox knockout mice, *Sal* saline, *U* U0126 (2 μg, i.c.v.), *Apo* apocynin (50 mg/kg, i.p.), *V or Veh* vehicle [10 % (*v*/*v*) DMSO] for U0126 or apocynin. Each value is the mean ± S.E.M. of six animals. ^*^ *P* < 0.05 vs. saline. ^**^ *P* < 0.01 vs. saline or vehicle/WT with saline. ^#^ *P* < 0.05 vs. vehicle/WT with MA [one-way ANOVA (**a** and **c**) or three-way ANOVA (**b** and **d**) was followed by Fisher’s LSD pairwise comparisons]

Significant decreases in MMP were observed 30 min (*P* < 0.05), 1 h (*P* < 0.05), 2 h (*P* < 0.01), 4 h (*P* < 0.05), and 6 h (*P* < 0.05) after MA in WT mice. The decrease was most pronounced 2 h post-MA. This decrease returned near control (saline-treated animal) level 1 day later (Fig. 3a). U0126 (*P* < 0.05), apocynin (*P* < 0.05), or genetic inhibition of p47phox (i.e., p47phox knockout mice) (*P* < 0.05) significantly protected the decrease in MMP 2 h post-MA. U0126 did not alter protection mediated by apocynin or genetic inhibition of p47phox (Fig. 3b).

MA treatment, however, significantly increased (*P* < 0.01) intramitochondrial calcium accumulation at the same time (2 h post-MA) in WT mice. The increase in intramitochondrial calcium accumulation returned near control (saline) level 1 day later (Fig. 3c). U0126 (*P* < 0.05), apocynin (*P* < 0.05), or genetic inhibition of p47phox (*P* < 0.05) significantly attenuated intramitochondrial calcium accumulation 2 h after MA. U0126 did not significantly affect attenuation produced by apocynin or p47phox knockout (Fig. 3d).

### ERK inhibitor U0126, apocynin, or genetic depletion of p47phox protects MA-induced oxidative stress and decreases in mitochondrial MnSOD expression; U0126 does not significantly affect the protection mediated by apocynin or p47phox knockout

We have shown that mitochondrial oxidative stress and impaired mitochondrial antioxidant system might mediate dopaminergic degeneration induced by multiple doses of MA [4]. We examined here whether a toxic dose of MA also significantly impairs MMP and intramitochondrial Ca^2+^ level and whether inhibition of ERK, PHOX, or p47phox gene modulates mitochondrial ROS and mitochondrial MnSOD (SOD-2) in the striatum of mice.

**Fig. 4:**
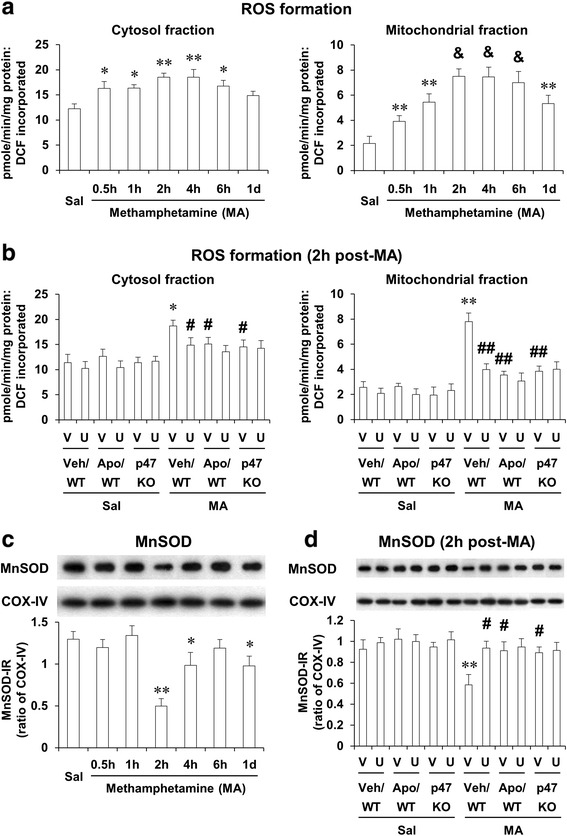
Effects of U0126, apocynin, or p47phox knockout on cytosolic and mitochondrial oxidative burdens after MA. Changes in cytosolic and mitochondrial reactive oxygen species (ROS) formation (**a**) and mitochondrial MnSOD expression (**c**) after MA treatment and effects of U0126, apocynin, or p47phox knockout on ROS (**b**) and MnSOD expression (**d**) 2 h after MA (35 mg/kg, i.p.). *WT* wild-type mice. *p47 KO* p47phox knockout mice, *Sal* saline, *U* U0126 (2 μg, i.c.v.), *Apo* apocynin (50 mg/kg, i.p.), *V or Veh* vehicle [10 % (*v*/*v*) DMSO] for U0126 or apocynin. Each value is the mean ± S.E.M. of six animals. ^*^ *P* < 0.05, ^**^ *P* < 0.01 vs. saline or vehicle/WT with saline. ^&^ *P* < 0.001 vs. saline. ^#^ *P* < 0.05, ^##^ *P* < 0.01 vs. vehicle/WT with MA [one-way ANOVA (**a** and **c**) or three-way ANOVA (**b** and **d**) was followed by Fisher’s LSD pairwise comparisons]

As shown in Fig. 4a, cytosolic ROS was significantly increased 30 min (*P* < 0.05), 1 h (*P* < 0.05), 2 h (*P* < 0.01), 4 h (*P* < 0.01), and 6 h (*P* < 0.05) after MA. In contrast, mitochondrial ROS was also significantly increased 30 min (*P* < 0.01), 1 h (*P* < 0.01), 2 h (*P* < 0.001), 4 h (*P* < 0.001), 6 h (*P* < 0.001), and 1 day (*P* < 0.01) after MA in WT mice (Fig. 4a), indicating that ROS formation is more pronounced in mitochondrial fraction than that in cytosolic fraction.

We then investigated the effect of U0126, apocynin, or genetic inhibition of p47phox (using p47phox KO mice) on the cytosolic and mitochondrial ROS formation 2 h post-MA. Attenuation of cytosolic ROS formation by U0126 (*P* < 0.05), apocynin (*P* < 0.05), or p47phox knockout (*P* < 0.05 vs. vehicle/WT) was observed in the striatum of mice. The attenuation by U0126 (*P* < 0.01), apocynin (*P* < 0.01), or p47phox knockout (*P* < 0.01) against mitochondrial ROS formation appeared to be more evident than cytosolic ROS formation. Consistently, MA-induced formation of ROS parallels that of 4-hydroxynonenal (HNE) or protein carbonyl. U0126, apocynin, or p47phox knockout also revealed protective effects against MA-induced formation of HNE and protein carbonyl (Additional file 1; Additional file 2: Figs. S6 and S7).

In contrast, mitochondrial MnSOD (SOD-2) expression was decreased significantly 2 h (*P* < 0.01), 4 h (*P* < 0.05), and 1 day (*P* < 0.05) post-MA. U0126 (*P* < 0.05), apocynin (*P* < 0.05), or p47phox knockout (*P* < 0.05) significantly recovered reduced SOD-2 expression 2 h after MA. Importantly, U0126 did not show any additional protection against antioxidant potentials (Fig. 4b) and enhanced SOD-2 expression mediated by apocynin or p47phox knockout (Fig. 4c, d).

### ERK inhibitor U0126, apocynin, or genetic depletion of p47phox protects MA-induced increases in mitochondrial translocation of cleaved PKCδ; U0126 does not significantly affect the protection mediated by apocynin or p47phox knockout

Recently, we have proposed that mitochondrial translocation of cleaved PKCδ plays a critical role in pro-apoptosis induced by MA [4, 8, 63]. We examined the effect of the inhibition of ERK, PHOX, or p47phox gene against mitochondrial translocation of cleaved PKCδ induced by a toxic dose of MA in the striatum of mice.

**Fig. 5:**
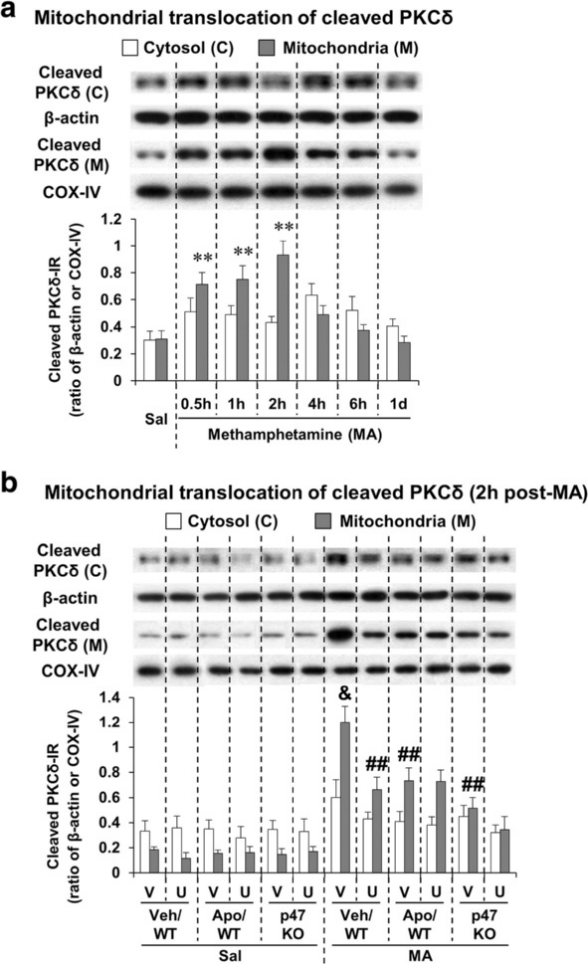
Effects of U0126, apocynin, or p47phox knockout on mitochondrial translocation of cleaved PKCδ after MA. Mitochondrial translocation of cleaved PKCδ after MA (35 mg/kg, i.p.) treatment (**a**) and effects of U0126, apocynin, or p47phox knockout on mitochondrial translocation of cleaved PKCδ (**b**). *WT* wild-type mice, *p47 KO* p47phox knockout mice, *Sal* saline, *U* U0126 (2 μg, i.c.v.), *Apo* apocynin (50 mg/kg, i.p.), *V or Veh* vehicle [10 % (*v*/*v*) DMSO] for U0126 or apocynin. Each value is the mean ± S.E.M. of six animals. ^**^ *P* < 0.01 vs. saline. ^&^ *P* < 0.001 vs. vehicle/WT with saline. ^##^ *P* < 0.01 vs. vehicle/WT with MA [one-way ANOVA (**a**) or three-way ANOVA (**b**) was followed by Fisher’s LSD pairwise comparisons]

As shown in Fig. 5, mitochondrial translocation of cleaved PKCδ did not change in the absence of MA. However, mitochondrial translocation of cleaved PKCδ was significantly increased 30 min (*P* < 0.01), 1 h (*P* < 0.01), and 2 h (*P* < 0.01) after MA treatment in WT mice (Fig. 5a). Since mitochondrial dysfunction (i.e., decrease in MMP and increase in intramitochondrial calcium accumulation) was most evident 2 h post-MA, we examined effect of U0126, apocynin, or genetic inhibition of p47phox (using p47phox KO mice) at that time against mitochondrial translocation of cleaved PKCδ (Fig. 5b). U0126 (*P* < 0.01), apocynin (*P* < 0.01), or genetic inhibition of p47phox (*P* < 0.01) significantly protected MA-induced mitochondrial translocation of cleaved PKCδ. However, U0126 did not affect the protection mediated by apocynin or p47phox knockout (Fig. 5b).

### ERK inhibitor U0126, apocynin, or genetic depletion of p47phox protects MA-induced increases in Iba-1-labeled microglia in the striatum of mice; U0126 does not significantly affect the protection mediated by apocynin or p47phox knockout

**Fig. 6:**
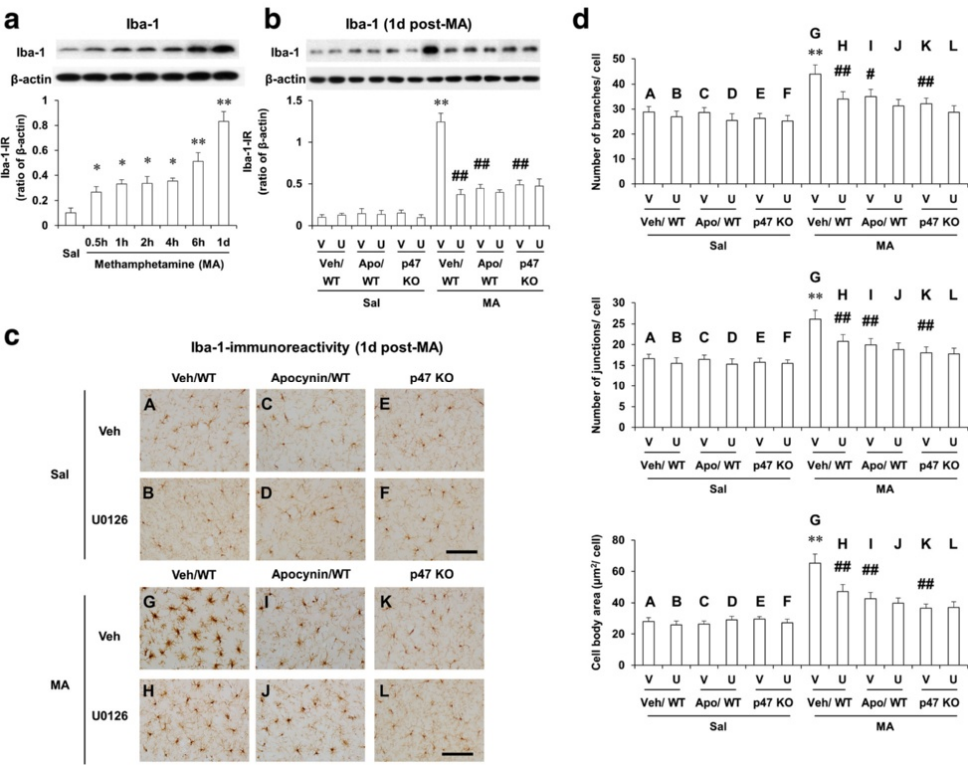
Effects of U0126, apocynin, or p47phox knockout on Iba-1 expression after MA. Changes in Iba-1 expression after MA treatment (**a**) and effects of U0126, apocynin, or p47phox knockout on Iba-1 expression (**b**) and microglial activation as labeled by Iba-1 (**c**, **d**) 1 day after MA (35 mg/kg, i.p.). *WT* wild-type mice, *p47 KO* p47phox knockout mice, *Sal* saline, *U or U0126* U0126 (2 μg, i.c.v.), *Apo* apocynin (50 mg/kg, i.p.), *V or Veh* vehicle [10 % (*v*/*v*) DMSO] for U0126 or apocynin. Each value is the mean ± S.E.M. of six animals. ^*^ *P* < 0.05, ^**^ *P* < 0.01 vs. saline or vehicle/WT with saline. ^#^ *P* < 0.05, ^##^ *P* < 0.01 vs. vehicle/WT with MA [one-way ANOVA (**a**) or three-way-ANOVA (**b**, **d**) was followed by Fisher’s LSD pairwise comparisons]. *Scale bar* = 100 μm

Accumulating evidence suggests that mitochondrial dysfunction links inflammation to neuronal death [32, 64]. Importantly, it has been proposed that microglia participate in neuroinflammation-associated activated MA intoxication [4, 8, 65–67]. Thus, we examined changes in Iba-1 expression after MA. Initial increase (*P* < 0.05) was observed 30 min post-MA. This increase was strongly activated (*P* < 0.01) 1 day after MA (Fig. 6a). We examined the effect of U0126, apocynin, or genetic inhibition of p47phox (using p47phox KO mice) on Iba-1 expression 1 day after MA. U0126, apocynin, or genetic inhibition of p47phox significantly inhibited (*P* < 0.01) Iba-1 expression 1 day after MA (Fig. 6b). U0126 did not alter the inhibition by apocynin or genetic inhibition of p47phox.

Iba-1-IR by immunocytochemical analysis was comparable to Iba-1 expression by Western blot analysis. Significant microglial activation was observed 1 day after MA treatment, as revealed by cell skeleton analysis and cell size analysis (Additional file 1; Additional file 2: Fig. S8). MA treatment significantly increased the number of branches (*P* < 0.01), the number of junctions (*P* < 0.01), the number of triple points (*P* < 0.01), the summed branch length (*P* < 0.05), cell size (*P* < 0.01), cell body size (*P* < 0.01), and cell body to cell size ratio (*P* < 0.05), but did not significantly alter average branch length (Additional file 1; Additional file 2: Fig. S8). Then, we examined the effect of U0126, apocynin, or genetic inhibition of p47phox on the microglial activation induced by MA. U0126, apocynin, or p47phox gene knockout significantly attenuated MA-induced increases in the number of branches (U0126 or p47phox KO, *P* < 0.01; apocynin, *P* < 0.01), the number of junctions (U0126, apocynin, or p47phox KO, *P* < 0.01), and cell body size (U0126, apocynin, or p47phox KO, *P* < 0.01). U0126 did not affect the attenuation mediated by apocynin or p47phox gene knockout (Fig. 6c, d).

### ERK inhibitor U0126, apocynin, or genetic depletion of p47phox attenuates MA-induced microglial differentiation into M1 type in the striatum of mice; U0126 does not significantly affect the attenuation mediated by apocynin or p47phox knockout

**Fig. 7:**
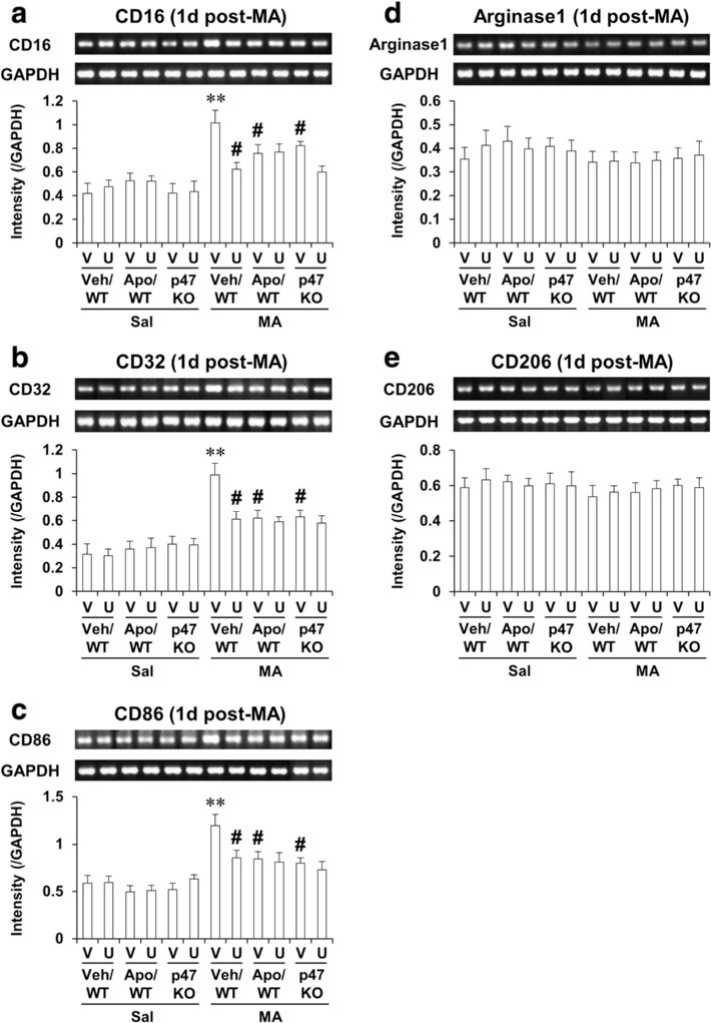
Effects of U0126, apocynin, or p47phox knockout on microglial differentiation 1 day after MA. Microglial differentiation into M1 type (**a**–**c**) and into M2 type (**d**–**e**). Gene primer sequences for RT-PCR analysis were shown in Table 1. *WT* wild-type mice, *p47 KO* p47phox knockout mice, *Sal* saline, *U* U0126 (2 μg, i.c.v.), *Apo* apocynin (50 mg/kg, i.p.), *V* or *Veh* vehicle [10 % (*v*/*v*) DMSO] for U0126 or apocynin. Each value is the mean ± S.E.M. of six animals. ^**^ *P* < 0.01 vs. vehicle/WT with saline. ^#^ *P* < 0.05 vs. vehicle/WT with MA (three-way ANOVA was followed by Fisher’s LSD pairwise comparisons)

It has been suggested that macrophages/microglia play different roles in tissue repair or damage in response to central nervous system (CNS) injury. These divergent effects may be due to distinct macrophage/microglial subsets, i.e., “classically activated” pro-inflammatory (M1) or “alternatively activated” anti-inflammatory (M2) cells [68–70]. The mRNA level of M1 markers were significantly enhanced (CD16, CD32, or CD86; *P* < 0.01 vs. corresponding vehicle) 1 day after MA treatment. This enhancement was significantly inhibited (*P* < 0.05) by U0126, apocynin, or p47phox knockout (Fig. 7a–c). Importantly, U0126 did not significantly affect the inhibition by apocynin or p47phox knockout (Fig. 7a–c). The mRNA levels of M2 markers (arginase 1 and CD206) appeared to be reduced without reaching statistical significance. U0126, apocynin, or p47phox knockout also did not significantly alter the levels of M2 markers (Fig. 7d, e).

### ERK inhibitor U0126, apocynin, or p47phox antisense oligonucleotide attenuates MA-induced increases in TUNEL-positive cells, cytosolic release of cytochrome c, and cleaved caspase 3 expression in the striatum of Taconic ICR mice; U0126 does not significantly affect the attenuation mediated by apocynin or p47phoxASO

**Fig. 8:**
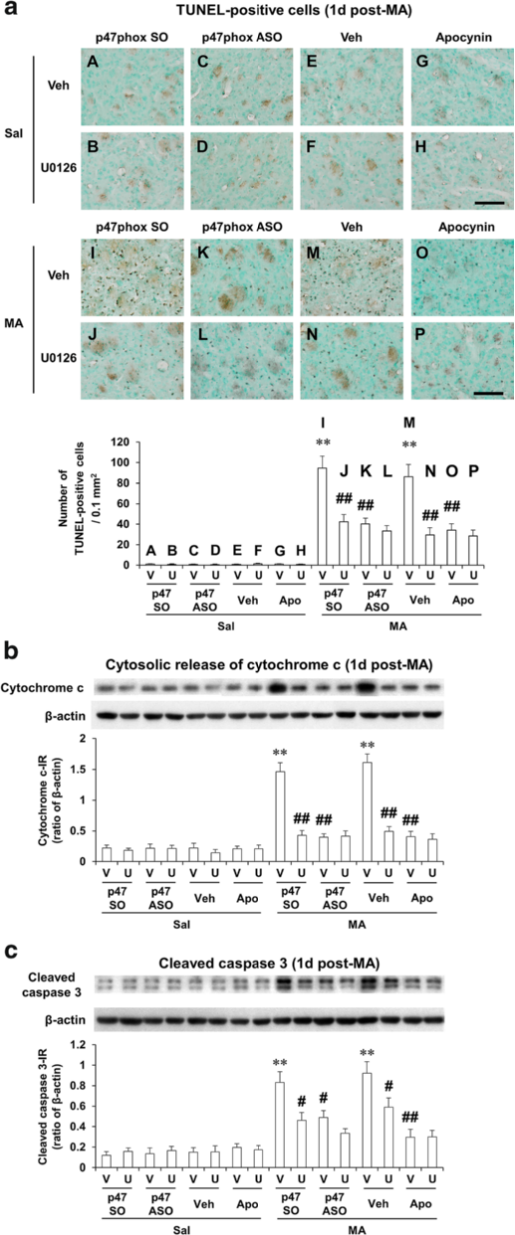
Effects of U0126, apocynin, or p47phox knockout on pro-apoptotic changes after MA. Effects of U0126, apocynin, or p47phox gene knockout on TUNEL-positive cells (**a**), cytosolic release of cytochrome c (**b**), and cleaved caspase 3 expression (**c**) 1 day after MA (35 mg/kg, i.p.) in the Taconic ICR mice. *p47 SO or p47phox SO* p47phox sense oligonucleotide, *p47 ASO or p47phox ASO* p47phox antisense oligonucleotide, *Sal* saline, *U or U0126* U0126 (2 μg, i.c.v.), *Apo* apocynin (50 mg/kg, i.p.), *V or Veh* vehicle [10 % (*v*/*v*) DMSO] for U0126 or apocynin. Each value is the mean ± SEM of six animals. ^**^ *P* < 0.01 vs. respective saline-group. ^#^ *P* < 0.05, ^##^ *P* < 0.01 vs. vehicle/p47phox SO with MA or vehicle/vehicle with MA (three-way ANOVA was followed by Fisher’s LSD pairwise comparisons). *Scale bar* = 100 μm

As we [4, 8, 9] and others [15, 16] have reported, it is well-recognized that Taconic ICR mice are sensitive to MA-induced TUNEL-positive reaction. As 1 day post-MA is most sensitive to TUNEL-positive reaction [4, 8, 9, 15, 16], we also evaluated other parameters (i.e., cytosolic release of cytochrome c and cleaved caspase 3) at the same time. In the absence of MA, TUNEL-positive cells were barely observed. They were increased significantly (*P* < 0.01) 1 day after a toxic dose of MA (35 mg/kg. i.p.). This significant increase [with p47phox sense oligonucleotide (SO) or vehicle] in the Taconic ICR mice was significantly inhibited (*P* < 0.01) by U0126, apocynin, or genetic inhibition of p47phox [i.e., p47phox antisense oligonucleotide (ASO)] in Taconic ICR mice (Fig. 8a).

In the absence of MA, no significant changes were observed in the cytosolic release of cytochrome c (Fig. 8b) and cleaved caspase 3 expression (Fig. 8c). MA treatment significantly increased these parameters consistently in Taconic ICR mice. MA-induced significant increases in cytosolic release of cytochrome c, and cleaved caspase 3 were significantly attenuated by U0126, apocynin, or p47phox ASO. Consistently, these increases were significantly inhibited by U0126, apocynin, or p47phox ASO (Fig. 8b, c). However, U0126 did not significantly affect the inhibition mediated by apocynin or p47phox ASO (Fig. 8a–c).

### ERK inhibitor U0126, apocynin, or p47phox knockout protects MA-induced decreases in tyrosine hydroxylase expression, TH-immunoreactivity and dopamine level and increase in dopamine turnover rate in the striatum of mice; U0126 does not significantly affect the protection mediated by apocynin or p47phox knockout

Recent reports demonstrated that a single high MA dose (30 mg/kg, i.p.) produces persistent monoaminergic deficits [71], including dopaminergic impairments [72]. We examined the role of ERK, PHOX, or p47phox gene in the dopaminergic loss induced by a single dose of MA (35 mg/kg, i.p.).

**Fig. 9:**
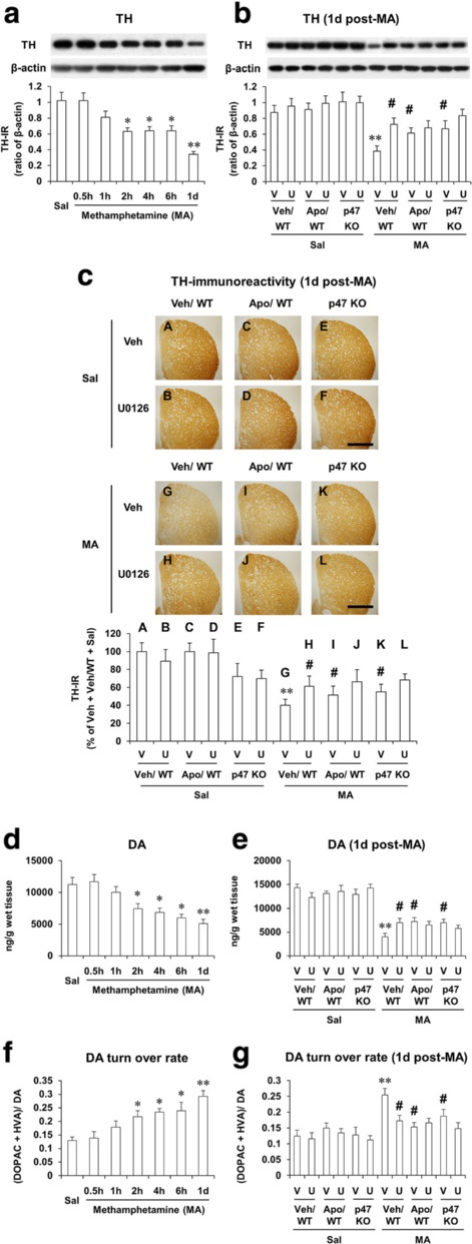
Effects of U0126, apocynin, or p47phox knockout on dopaminergic impairments after MA. Changes in TH expression (**a**), dopamine level (**d**), and dopamine turnover rate (**f**) after MA treatment and effects of U0126, apocynin, or p47phox gene knockout on TH expression (**b**), TH-immunoreactivity (**c**), dopamine level (**e**), and dopamine turnover rate (**g**). *WT* wild-type mice, *p47 KO* p47phox knockout mice, *Sal* saline, *U or U0126* U0126 (2 μg, i.c.v.), *Apo* apocynin (50 mg/kg, i.p.), *V or Veh* vehicle [10 % (*v*/*v*) DMSO] for U0126 or apocynin. Each value is the mean ± S.E.M. of six animals. ^*^ *P* < 0.05, ^**^ *P* < 0.01 vs. saline or vehicle/WT with saline. ^#^ *P* < 0.05 vs. vehicle/WT with MA [one-way ANOVA (**a**, **d**, and **f**) or three-way ANOVA (**b**, **c**, **e**, and **g**) was followed by Fisher’s LSD pairwise comparisons]. *Scale bar* = 1 mm

As shown in Fig. 9, TH expression (Fig. 9a) and dopamine level (Fig. 9d) were significantly decreased 2 h (*P* < 0.05), 4 h (*P* < 0.05), 6 h (*P* < 0.05), and 1 day (*P* < 0.01) after MA, respectively. The decrease of TH expression was comparable to that of dopamine in our model (Fig. 9a, d). However, the dopamine turnover rate was significantly increased over time (Fig. 9f). U0126, apocynin, or p47phox knockout significantly attenuated reductions in TH expression (Fig. 9b), TH-immunoreactivity (TH-IR) (Fig. 9c), and dopamine level (Fig. 9d) 1 day after MA. Consistently, MA-induced increase in dopamine turnover rate was significantly attenuated by U0126, apocynin, or p47phox knockout (Fig. 9g). However, U0126 did not influence protective activities afforded by apocynin or p47phox knockout (Fig. 9b, c, e, g).

## Discussion

Previous studies have shown that activation of PHOX activity requires p47phox phosphorylation, a protein that plays an important role in the translocation of cytosolic components to cytochrome b558, as well as in the assembly and activation of PHOX [36, 51, 52, 73]. Phosphorylation of p47phox constitutes one of the key intracellular events associated with PHOX activation, and Ser345 phosphorylation of p47phox by the MAPK protein ERK plays a critical role in the potentiation of PHOX activation by pro-inflammatory agents [36, 51]. Therefore, we first examined the levels of MA-induced p47phox phosphorylation using the anti-phospho-Ser345-p47phox antibody.

Two prominent features of this protective role of apocynin or p47phox depletion were observed in this study: (1) apocynin-mediated inhibition of p47 translocation is mediated through the inhibition of PHOX subunit p47phox phosphorylation at Ser345 mainly via suppression of the ERK-signaling pathway, and (2) apocynin attenuates MA-mediated oxidative stress (mitochondrial > cytosolic fraction), mitochondrial dysfunction, microglial activation (towards M1 phenotype), pro-apoptosis, and dopaminergic loss mainly through the inhibition of ERK-dependent p47phox activation.

Participation of ERK1/2 in the activation of PHOX was also proven by a previous study using microglial cells/rat primary mesencephalic neuron-glia cultures stimulated with lipopolysaccharides (LPS) [36]. The fact that apocynin significantly inhibits the formation of ROS (oxidative damage) 2 h after MA stimulation led us to examine this factor in greater detail by using p47phox-deficient mice. The findings that apocynin could significantly lessen the MA-induced dopaminergic loss in WT, but has no significant effect in response to p47phox knockout mice, suggest that the protective effect of apocynin is most likely mediated through the inhibition of p47phox activity.

Translocation of p47phox, p67phox, p40phox, and rac2 to the plasma membrane are required for the activation of PHOX [73]. The phosphorylation of Ser345 of p47phox by pro-inflammatory agents enhances this translocation event [36, 51, 52]. While investigating the mechanism by which apocynin inhibits PHOX activity, we found that apocynin significantly inhibits this MA-induced p47phox phosphorylation at Ser345, resulting in the inhibition of p47phox translocation. As Ser345 is located in the MAPK consensus sequence [51], we examined whether apocynin inhibits components of the MAPK-signaling pathway, and our results indicate that apocynin shows a significant inhibitory effect on MA-induced ERK phosphorylation. However, MA-induced induction in p38- or JNK-phosphorylation was much less pronounced than in ERK phosphorylation. Furthermore, a specific ERK inhibitor U0126 exhibited strong inhibitory effects against MA-induced p47phox phosphorylation, p47phox translocation, oxidative damage, pro-apoptosis, and neurodegeneration, suggesting a central role of ERK in these effects. These findings, coupled with previous findings on the role of ERK in PHOX activation [36], strongly indicate that it is ERK that regulates p47phox phosphorylation and constitutes the crucial target for apocynin-mediated inhibition of PHOX activation.

A previous study demonstrated that disturbed Ca^2+^ homeostasis may mediate dopaminergic degeneration, such as PD [74] and MA intoxication [4, 8]. We examined here whether genetically inhibiting p47phox would affect MMP and intramitochondrial Ca^2+^ accumulation in the striatum of mice. Accumulating evidence suggests that mitochondrial damage links inflammation to neuronal death [4, 8, 75]. Moreover, it is recognized that the role of glial cells in MA-induced neurotoxicity is essential to identify factors contributing to, or mitigating, MA-induced damage to DA nerve terminals [4, 21, 65–67, 76–78]. Importantly, it has been proposed that microglia participate in neurotoxicity associated with MA intoxication [4, 21, 65–67, 78].

We were interested in whether or not apocynin would affect this apoptotic signaling pathway after MA exposure. An earlier report demonstrated that MA induces apoptotic cell death in striatal neurons [13]. In the present study, we chose TUNEL staining (which labels the occurrence of DNA fragmentation, which occurs late in apoptosis). We previously failed to observe MA-induced TUNEL-positive cells in the striatum of C57BL/6 mice 12 h, 1 day, or 3 days after the final MA administration (i.e., four injections of 7 mg/kg MA, intraperitoneally at 2 h intervals or a single injection of MA 20–40 mg/kg), suggesting that the C57BL/6 background is not sensitive to TUNEL staining [9, 77]. Thus, according to previous reports [9, 15, 16], we used 10-week-old male Taconic ICR mice. Because apoptotic cell death was detectable at 20 mg/kg MA and reached a significant level at 35 mg/kg in our previous study, a 35-mg/kg dose of MA was chosen for the present study. We also sacrificed animals 1 day after MA administration [4, 8, 9, 15, 16], and TUNEL-positive cells were induced maximally at this time point.

The relationship between mitochondrial damage, oxidative stress, and neuronal dysfunction has been recognized by the effects of excessive production of ROS within mitochondria, which leads to a reduction of mitochondrial antioxidant activity, in turn causing impairment of mitochondrial function [4, 8, 79, 80]. Our results clearly indicate that MA-induced toxic damage is more pronounced in the mitochondrial fraction than in the cytosolic fraction in WT mice, and that apocynin or genetically inhibiting p47phox significantly attenuates this oxidative damage, mitochondrial dysfunction, pro-apoptotic changes, and dopaminergic impairment. We demonstrated that PKCδ is an oxidative stress-sensitive kinase, and its activation via caspase-3-dependent proteolysis induces apoptotic cell death in MA-induced dopaminergic toxicity [4, 63]. Therefore, the protective effect of apocynin against MA-induced PKCδ activation and dopaminergic deficits might reflect an anti-peroxidative (mitochondrial > cytosolic) potential by targeting p47phox gene. Indeed, apocynin does not significantly alter neuroprotective activity mediated by p47phox gene knockout, suggesting that p47phox is a critical target for the neuroprotective activity of apocynin. To the best of our knowledge, the current study is the first to investigate the role of p47phox per se in apocynin-mediated neuroprotective potential with recovery of mitochondrial function.

We propose here that MA potentiates mitochondrial oxidative stress and also impairs the mitochondrial detoxification system, and MMP, possibly due to Ca^2+^ accumulation. Increased intracellular Ca^2+^ promotes the accumulation of Ca^2+^ within the mitochondrial matrix when total Ca^2+^ uptake exceeds total Ca^2+^ efflux from mitochondria [81]. Mitochondrial Ca^2+^ overload may also lead to the uncoupling of mitochondrial electron transport and may potentiate oxidative stress. Decreases in MMP and increases in oxidative damage after MA treatment could be mediated by Ca^2+^ entry.

Based on the importance of MnSOD in our experimental condition, we sought to determine whether or not this mitochondrial enzyme would provide neuroprotection against MA neurotoxicity. It has been acknowledged that MnSOD overexpression attenuates dopaminergic toxicity induced by MA [82] or 1-methyl-4-phenyl-1,2,3,6-tetrahydropyridine (MPTP) [83] and protects cells from apoptosis [84]. Maragos et al. [82] demonstrated that the formation of protein carbonyls is less pronounced in MnSOD transgenic overexpressing (Tg) mice than that in non-Tg mice against MA toxicity. Furthermore, a previous report indicated that increased MnSOD expression without a change in Cu/Zn-SOD, catalase, or glutathione peroxidase activities [85] conferred neuroprotection against dopamine loss in a model of neuronal damage, indicating a possible role for detoxification of MA-induced ROS by scavenging of superoxide radicals in the mitochondria. Interestingly, MnSOD overexpression failed to protect MA-induced reductions in 5-hydroxytryptamine (5-HT) and 5-hydroxyindoleacetic acid (5-HIAA) [82], suggesting that the mitochondrial mechanism may not be involved in serotonergic toxicity. Our results indicate that higher levels of MnSOD might be associated with enhanced mitochondrial maintenance and could contribute to reducing apoptosis that has been induced by mitochondrial damage in the condition with dopaminergic impairments.

Microglial activation and oxidative stress induced by mitochondrial toxins (i.e., 3-nitropropionic acid) caused neuronal loss in the striatum [64]. Mitochondria can be a target of free radical stress initiated by activated microglia. The combination of mitochondrial dysfunction, oxidative stress, and exacerbated activation of microglia generates a cycle that appears to lead to progressive dopaminergic neuronal cell death [86]. We raise the possibility that apocynin might, at least in part, block Ca^2+^ entry through the mitochondrial translocation of p47phox, given that apocynin primarily attenuated mitochondrial dysfunction and mitochondrial ROS.

In our study, a toxic dose (35 mg/kg, i.p. × 1) of MA induced the transformation of ramified/resting microglia into reactive hypertrophic microglia, as evidenced by increases in the number of branches and cell body size. Based on morphological characteristics, microglia can be classified into at least four stages of activation: (1) ramified/resting, (2) hypertrophic, (3) bushy, and (4) amoeboid microglia [57, 87]. According to this classification, hypertrophic microglia have large cell bodies and long, thick, highly branched processes, whereas bushy or amoeboid microglia have fewer thick and rarely branched processes, even though cell bodies become larger than hypertrophic microglia. Raineri et al. [88] showed that a multiple dose regimen of MA (i.e., 5 mg/kg × 4) induces amoeboid, as well as hyper-ramified microglia, in mouse striatum; however, amoeboid microglia were rarely observed in a toxic dose regimen of MA in our study. Thus, this issue requires further exploration.

Current results are in line with our previous reports that MA treatment significantly increased the mRNA expression of M1 phenotypic markers (CD16, CD32, and CD86), suggesting that microglia after MA treatment existed primarily in the classically activated state [4, 21], which is pro-inflammatory. Thus, our results indicate that neuroprotection by apocynin or p47phox knockout is mediated by its anti-inflammatory properties.

We have reported that the oligomeric form of α-synuclein was obviously increased after MA [12]. Interestingly, earlier studies have shown that aggregated α-synuclein released into the extracellular space from dying or dead DA neurons can directly induce microglia towards M1 phenotype with the activation of NADPH oxidase, increasing production of ROS and pro-inflammatory cytokines [89–92]. Overexpression of mutant α-synuclein solely in microglia switches microglia into a more reactive M1 phenotype characterized by elevated levels of pro-inflammatory cytokines [93]. Similarly, typical characteristics of M1 phenotype, including the activation of PHOX as well as the release of various pro-inflammatory mediators, were observed in the MPTP-intoxicated models [94], indicating that this phenomenon, at least in part, parallels current results. However, although inhibition of PHOX or genetic inhibition of its functional p47phox subunit switches microglial activation from M1 to M2 in response to LPS challenge [95], either inhibition did not significantly alter the mRNA expression of M2 phenotypic markers induced by MA in this study. Similar to the current study, multiple doses of MA did not significantly decrease M2 phenotype markers in our previous study [4]. Thus, the interactive modulation between M1- and M2-activated populations remains to be determined [68–70].

## Conclusions

**Fig. 10:**
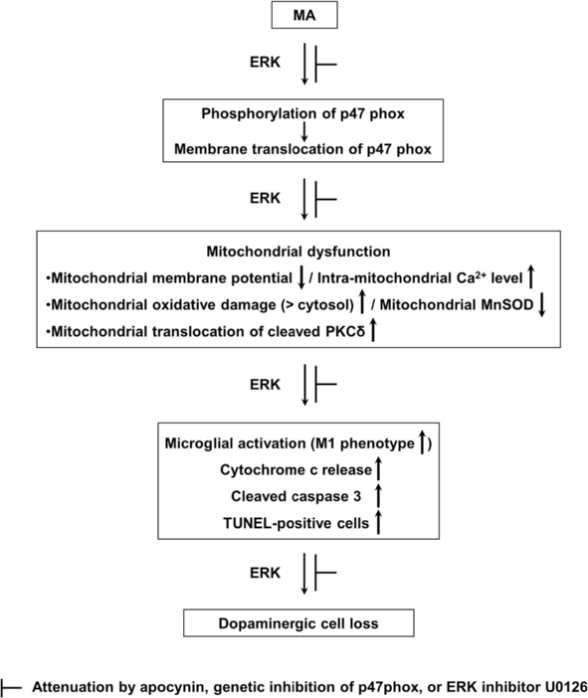
A schematic depiction of the role of PHOX in MA-induced ERK-dependent dopaminergic neurotoxicity. Treatment with a toxic dose of MA resulted in significant phosphorylations in ERK1/2 (> p38 and JNK), and p47phox followed by membrane translocation of p47phox in the striatum of mice. The activation of p47phox promotes mitochondrial stress followed by microglial activation into M1 phenotype, and pro-apoptotic process, and leading to dopaminergic impairments. These signalings were promoted by ERK1/2. ERK inhibitor U0126 did not exhibit any additional positive effect in response to the protection offered by apocynin or p47phox genetic inhibition, suggesting that ERK regulates p47phox activation, and thus ERK is the crucial target for apocynin-mediated inhibition of PHOX activation

We have shown in this study that a neurotoxic dose of MA-induced pro-apoptosis requires ERK-dependent p47phox activation followed by oxidative stress (mitochondria > cytosol), mitochondrial dysfunction, and pro-inflammatory changes (i.e., exacerbated activation of M1-type microglia). Thus, inhibition of ERK-dependent p47phox activation is critical for dopaminergic neuroprotective potential mediated by apocynin or p47phox knockout (Fig. 10).

## Additional files

Additional file 1:
**Supplemental information.** Supplemental materials and methods, supplemental results, supplemental references, and supplemental figure legends in detail. (DOCX 41 kb) Additional file 2:
**Supplemental figures. Fig. S1.** Experimental design I. Effect of inhibition of PHOX on the neurotoxicity induced by a multiple dose regimen (7 mg/kg, i.p. × 4; a) or a toxic dose regimen (35 mg/kg, i.p. × 1; b) of MA. **Fig. S2.** Effect of apocynin or p47phox knockout on the hyperthermia induced by the multiple doses (a) or a toxic dose (b) of MA. **Fig. S3. **Effect of apocynin or p47phox knockout on the decrease in tyrosine hydroxylase-immunoreactivity induced by multiple doses or a toxic dose of MA. **Fig. S4.** Effect of apocynin or p47phox knockout on the changes in dopamine level (a) and dopamine turnover rate (b) in the striatum induced by the multiple doses or a toxic dose of MA.**Fig. S5.** Experimental design II. The time-dependent alterations in experimental parameters after MA (35 mg/kg, i.p. × 1) treatment (a). Effect of U0126, apocynin, or p47phox knockout on the MA-induced neurotoxicity (b). **Fig. S6. **Cytosolic and mitochondrial changes in the level of 4-hydroxynonenal (HNE) adduct after the MA treatment (a), and the effect of U0126, apocynin, or p47phox knockout on the increase in HNE level 2 h after MA (35 mg/kg, i.p. × 1) (b).**Fig. S7. **Cytosolic and mitochondrial changes in the level of protein carbonyl after the MA treatment (a), and the effect of U0126, apocynin, or p47phox knockout on the increase in protein carbonyl level 2 h after MA (35 mg/kg, i.p. × 1) (b). **Fig. S8. **Morphological changes in microglia after MA treatment in the striatum. Morphological changes were determined by the analysis of cell skeleton (a, b) or cell body size (c, d). Detailed figure legends are included in the Additional file 1. (PDF 14030 kb)

## Abbreviations

ANOVA: analysis of variance
ASO: antisense oligonucleotide
Cleaved PKCδ: cleaved form of protein kinase C delta type
DA: dopamine
DMSO: dimethyl sulfoxide
DOPAC: 3,4-dihydroxyphenylacetic acid
EDTA: ethylenediaminetetraacetic acid
EGTA: ethylene glycol-bis(2-aminoethyl ether)-N,N,N′,N′-tetraacetic acid
ERK: extracellular signal-regulated kinase
HEPES: 4-(2-hydroxyethyl)-1-piperazineethanesulfonic acid
HNE: 4-hydroxynonenal
HVA: homovanillic acid
Iba-1: ionized calcium-binding adaptor molecule 1
JNK: c-Jun N-terminal kinase
KO: knockout
LPS: lipopolysaccharides
MA: methamphetamine
MAPKs: mitogen-activated protein kinases
MMP: mitochondrial membrane potential
MnSOD: manganese-dependent superoxide dismutase
MPTP: 1-methyl-4-phenyl-1,2,3,6-tetrahydropyridine
p38: p38 mitogen-activated protein kinase
PD: Parkinson’s disease
PHOX: NADPH oxidase
ROS: reactive oxygen species
RT-PCR: reverse transcription and polymerase chain reaction
SO: sense oligonucleotide
Tg: transgenic
TH: tyrosine hydroxylase
TH-IR: TH-immunoreactivity
TUNEL: terminal deoxynucleotidyl transferase dUDP nick-end labeling
WT: wild-type

## References

[1] Nakajima A, Yamada K, Nagai T, Uchiyama T, Miyamoto Y, Mamiya T (2004). Role of tumor necrosis factor-alpha in methamphetamine-induced drug dependence and neurotoxicity. J Neurosci.

[2] Walsh SL, Wagner GC (1992). Motor impairments after methamphetamine-induced neurotoxicity in the rat. J Pharmacol Exp Ther.

[3] Kim HC, Jhoo WK, Choi DY, Im DH, Shin EJ, Suh JH (1999). Protection of methamphetamine nigrostriatal toxicity by dietary selenium. Brain Res.

[4] Shin EJ, Shin SW, Nguyen TT, Park DH, Wie MB, Jang CG (2014). Ginsenoside Re rescues methamphetamine-induced oxidative damage, mitochondrial dysfunction, microglial activation and dopaminergic degeneration by inhibiting the protein kinase Cδ gene. Mol Neurobiol.

[5] Morrow BA, Roth RH, Redmond DE, Elsworth JD (2011). Impact of methamphetamine on dopamine neurons in primates is dependent on age: implications for development of Parkinson’s disease. Neuroscience.

[6] Cadet JL, Krasnova IN (2009). Molecular bases of methamphetamine-induced neurodegeneration. Int Rev Neurobiol.

[7] Giovanni A, Liang LP, Hasting TG, Zigmond MJ (1995). Estimating hydroxyl radical content in rat brain using systemic and intraventricular salicylate: impact of methamphetamine. J Neurochem.

[8] Nguyen XK, Lee J, Shin EJ, Dang DK, Jeong JH, Nguyen TT (2015). Liposomal melatonin rescues methamphatemine-elicited mitochondrial burdens, pro-apoptosis, and dopaminergic degeneration through the inhibition PKCδ gene. J Pineal Res.

[9] Shin EJ, Duong CX, Nguyen XK, Li Z, Bing G, Bach JH (2012). Role of oxidative stress in methamphetamine-induced dopaminergic toxicity mediated by protein kinase Cδ. Behav Brain Res.

[10] Krasnova IN, Cadet JL (2009). Methamphetamine toxicity and messengers of death. Brain Res Rev.

[11] Asanuma M, Miyazaki I, Higashi Y, Tsuji T, Ogawa N (2004). Specific gene expression and possible involvement of inflammation in methamphetamine-induced neurotoxicity. Ann N Y Acad Sci.

[12] Jung BD, Shin EJ, Nguyen XK, Jin CH, Bach JH, Park SJ (2010). Potentiation of methamphetamine neurotoxicity by intrastriatal lipopolysaccharide administration. Neurochem Int.

[13] Deng X, Cadet JL (2000). Methamphetamine-induced apoptosis is attenuated in the striata of copper-zinc superoxide dismutase transgenic mice. Mol Brain Res.

[14] Cadet JL, Krasnova IN, Jayanthi S, Lyles J (2007). Neurotoxicity of substituted amphetamines: molecular and cellular mechanisms. Neurotox Res.

[15] Zhu JP, Xu W, Angulo JA (2006). Methamphetamine-induced cell death: selective vulnerability in neuronal subpopulations of the striatum in mice. Neuroscience.

[16] Zhu JP, Xu W, Angulo N, Angulo JA (2006). Methamphetamine-induced striatal apoptosis in the mouse brain: comparison of a binge to an acute bolus drug administration. Neurotoxicology.

[17] Harvey DC, Lacan G, Melegan WP (2000). Regional heterogeneity of dopaminergic deficits in vervet monkey striatum and substantia nigra after methamphetamine exposure. Exp Brain Res.

[18] Kim HC, Jhoo WK, Shin EJ, Bing G (2000). Selenium deficiency potentiates methamphetamine-induced nigral neuronal loss; comparison with MPTP model. Brain Res.

[19] Kita T, Wagner GC, Nakashima T (2003). Current research on methamphetamine-induced neurotoxicity: animal models of monoamine disruption. J Pharmacol Sci.

[20] Sonsalla PK, Jochnowitz ND, Zeevalk GD, Oostveen JA, Hall ED (1996). Treatment of mice with methamphetamine produces cell loss in the substantia nigra. Brain Res.

[21] Wang Q, Shin EJ, Nguyen XK, Li Q, Bach JH, Bing G (2012). Endogenous dynorphin protects against neurotoxin-elicited nigrostriatal dopaminergic neuron damage and motor deficits in mice. J Neuroinflammation.

[22] Callaghan RC, Cunningham JK, Sajeev G, Kish SJ (2010). Incidence of Parkinson’s disease among hospital patients with metamphetamine-use disorders. Mov Disord.

[23] Callaghan RC, Cunningham JK, Sykes J, Kish SJ (2012). Increased risk of Parkinson’s disease in individuals hospitalized with conditions related to the use of methamphetamine or other amphetamine-type drugs. Drug Alcohol Depend.

[24] Curtin K, Fleckenstein AE, Robison RJ, Crookston MJ, Smith KR, Hanson GR (2015). Methamphetamine/amphetamine abuse and risk of Parkinson’s disease in Utah: a population-based assessment. Drug Alcohol Depend.

[25] Guilarte TR (2001). Is methamphetamine abuse a risk factor in parkinsonism?. Neurotoxicology.

[26] Wilson JM, Kalasinsky KS, Levey AI, Bergeron C, Reiber G, Anthony RM (1996). Striatal dopamine nerve terminal markers in human, chronic methamphetamine users. Nat Med.

[27] Wilson JM, Levey AI, Rajput A, Ang L, Guttman M, Shannak K (1996). Differential changes in neurochemical markers of striatal dopamine nerve terminals in idiopathic Parkinson’s disease. Neurology.

[28] Zhong XH, Haycock JW, Shannak K, Robitaille Y, Fratkin J, Koeppen AH (1995). Striatal dihydroxyphenylalanine decarboxylase and tyrosine hydroxylase protein in idiopathic Parkinson’s disease and dominantly inherited olivopontocerebellar atrophy. Mov Disord.

[29] Gluck MR, Moy LY, Jayatilleke E, Hogan KA, Manzino L, Sonsalla PK (2001). Parallel increases in lipid and protein oxidative markers in several mouse brain regions after methamphetamine treatment. J Neurochem.

[30] Jayanthi S, Ladenheim B, Cadet JL (1998). Methamphetamine-induced changes in antioxidant enzymes and lipid peroxidation in copper/zinc-superoxide dismutase transgenic mice. Ann N Y Acad Sci.

[31] Kobeissy FH, Warren MW, Ottens AK, Sadasivan S, Zhang Z, Gold MS (2008). Psychoproteomic analysis of rat cortex following acute methamphetamine exposure. J Proteome Res.

[32] Kroller-Schon S, Steven S, Kossmann S, Scholz A, Daub S, Oelze M (2014). Molecular mechanisms of crosstalk between mitochondria and NADPH oxidase through reactive oxygen species-studies in white blood cells and in animal models. Antioxid Redox Signal.

[33] Babior BM (1999). NADPH oxidase: an update. Blood.

[34] Vignais PV (2002). The superoxide-generating NADPH oxidase: structural aspects and activation mechanism. Cell Mol Life Sci.

[35] Gao HM, Zhou H, Hong JS (2012). NADPH oxidases: novel therapeutic targets for neurodegenerative diseases. Trends Pharmacol Sci.

[36] Qian L, Wei SJ, Zhang D, Hu X, Xu Z, Wilson B (2008). Potent anti-inflammatory and neuroprotective effects of TGF-β1 are mediated through the inhibition of ERK and p47phox-Ser345 phosphorylation and translocation in microglia. J Immunol.

[37] Wang Q, Chu CH, Qian L, Chen SH, Wilson B, Oyarzabal E (2014). Substance P exacerbates dopaminergic neurodegeneration through neurokinin-1 receptor-independent activation of microglial NADPH oxidase. J Neurosci.

[38] Wang Q, Chu CH, Oyarzabal E, Jiang L, Chen SH, Wilson B (2014). Subpicomolar diphenyleneiodonium inhibits microglial NADPH oxidase with high specificity and shows great potential as a therapeutic agent for neurodegenerative diseases. Glia.

[39] Abramov AY, Canevari L, Duchen MR (2004). β-amyloid peptides induce mitochondrial dysfunction and oxidative stress in astrocytes and death of neurons through activation of NADPH oxidase. J Neurosci.

[40] Gao HM, Liu B, Zhang W, Hong JS (2003). Critical role of microglial NADPH oxidase-derived free radicals in the in vitro MPTP model of Parkinson’s disease. FASEB J.

[41] Block ML, Hong JS (2005). Microglia and inflammation-mediated neurodegeneration: multiple triggers with a common mechanism. Prog Neurobiol.

[42] Block ML, Zecca L, Hong JS (2007). Microglia-mediated neurotoxcity: uncovering the molecular mechanisms. Nat Rev Neurosci.

[43] Gao HM, Hong JS (2008). Why neurodegenerative diseases are progressive: uncontrolled inflammation drives disease progression. Trends Immunol.

[44] Wang Q, Qian L, Chen SH, Chu CH, Wilson B, Oyarzabal E (2015). Post-treatment with ultra-low dose of NADPH oxidase inhibitor diphenyleneiodonium attenuates disease progression in multiple Parkinson’s disease models. Brain.

[45] Zhang W, Shin EJ, Wang T, Lee PH, Pang H, Wie MB (2006). 3-Hydroxymorphinan, a metabolite of dextromethorphan, protects nigrostriatal pathway against MPTP-elicited damage both in vivo and in vitro. FASEB J.

[46] Qin L, Liu Y, Hong JS, Crews FT (2013). NADPH oxidase and aging drive microglial activaton, oxidative stress, and dopaminergic neurodegeneration following systemic LPS administration. Glia.

[47] Qin L, Liu Y, Wang T, Wei SJ, Block ML, Wilson B (2004). NADPH oxidase mediates lipopolysaccharide-induced neurotoxicity and proinflammatory gene expression in activated microglia. J Biol Chem.

[48] Johnson DK, Schillinger KJ, Kwait DM, Hughes CV, McNamara EJ, Ishmael F (2002). Inhibition of NADPH oxidase activation in endothelial cells by ortho-methoxy-substituted catechols. Endothelium.

[49] Miller DK, Oelrichs CE, Sun GY, Simonyi A (2014). Subchronic apocynin treatment attenuates methamphetamine-induced dopamine release and hyperactivity in rats. Life Sci.

[50] Park M, Hennig B, Toborek M (2012). Methamphetamine alters occludin expression via NADPH oxidase-induced oxidative insult and intact caveolae. J Cell Mol Med.

[51] Dang PM, Stensballe A, Boussetta T, Raad H, Dewas C, Kroviarski Y (2006). A specific p47phox-serine phosphorylated by convergent MAPKs mediates neutrophil NADPH oxidase priming at inflammatory sites. J Clin Invest.

[52] El-Benna J, Dang PM, Gougerot-Pocidalo MA (2008). Priming of the neutrophil NADPH oxidase activation: role of p47phox phosphorylation and NOX2 mobilization to the plasma membrane. Semin Immunopalthol.

[53] Ni CW, Kumar S, Ankeny CJ, Jo H (2014). Development of immortalized mouse aortic endothelial cell lines. Vascular Cell.

[54] Fernandez SM, Lewis MC, Pechenino AS, Harburger LL, Orr PT, Gresack JE (2008). Estradiol-induced enhancement of object memory consolidation involves hippocampal extracellular signal-regulated kinase activation and membrane-bound estrogen receptors. J Neurosci.

[55] Franklin KBJ, Paxinos G (2008). The mouse brain in stereotaxic coordinates.

[56] Zhang D, Hu X, Wei SJ, Liu J, Gao H, Qian L (2008). Squamosamide derivative FLZ protects dopaminergic neurons against inflammation-mediated neurodegeneration through the inhibition of NADPH oxidase activity. J Neuroinflammation.

[57] Soltys Z, Orzylowska-Sliwinska O, Zaremba M, Orlowski D, Piechota M, Fiedorowicz A (2005). Quantitative morphological study of microglial cells in the ischemic rat brain using principal component analysis. J Neurosci Methods.

[58] Morrison HW, Filosa JA (2013). A quantitative spatiotemporal analysis of microglia morphology during ischemic stroke and reperfusion. J Neuroinflammation.

[59] Astafurov K, Elhawy E, Ren L, Dong CQ, Igboin C, Hyman L (2014). Oral microbiome link to neurodegeneration in glaucoma. PLoS One.

[60] Nemeth CL, Reddy R, Bekhbat M, Bailey J, Neigh GN (2014). Microglial activation occurs in the absence of anxiety-like behavior following microembolic stroke in female, but not male, rats. J Neuroinflammation.

[61] Hovens IB, Nyakas C, Schoemaker RG (2014). A novel method for evaluating microglial activation using ionized calcium-binding adaptor protein-1 staining: cell body to cell size ratio. Neuroimmunol Neuroinflammation.

[62] Marschallinger J, Schäffner I, Klein B, Gelfert R, Rivera FJ, Illes S (2015). Structural and functional rejuvenation of the aged brain by an approved anti-asthmatic drug. Nat Commun.

[63] Nam Y, Wie MB, Shin EJ, Nguyen TT, Nah SY, Ko SK (2015). Ginsenoside Re protects methamphetamine-induced mitochondrial burdens and proapoptosis via genetic inhibition of protein kinase C δ in human neuroblastoma dopaminergic SH-SY5Y cell lines. J Appl Toxicol.

[64] Ryu JK, Nagai A, Kim J, Lee MC, McLarnon JC, Kim SU (2003). Microglial activation and cell death induced by the mitochondrial toxin 3-nitropropionic acid: in vitro and in vivo studies. Neurobiol Dis.

[65] Friend DM, Keefe KA (2013). Glial reactivity in resistance to methamphetamine-induced neurotoxicity. J Neurochem.

[66] Thomas DM, Walker PD, Benjamins JA, Geddes TJ, Kuhn DM (2004). Methamphetamine neurotoxicity in dopamine nerve endings of the striatum is associated with microglial activation. J Pharmacol Exp Ther.

[67] Thomas DM, Kuhn DM (2005). Attenuated microglial activation mediates tolerance to the neurotoxic effects of methamphetamine. J Neurochem.

[68] Franco R, Fernández-Suárez D (2015). Alternatively activated microglia and macrophages in the central nervous system. Prog Neurobiol.

[69] Martinez FO, Gordon S. The M1 and M2 paradigm of macrophage activation: time for reassessment. F1000Prime Rep. 2014;6:13. 10.12703/P6-13 PMC3944738 24669294

[70] Moehle MS, West AB (2015). M1 and M2 immune activation in Parkinson’s disease: foe and ally?. Neuroscience.

[71] Silva CD, Neves AF, Dias AI, Freitas HJ, Mendes SM, Pita I (2014). A single neurotoxic dose of methamphetamine induces a long-lasting depressive-like behavior in mice. Neurotox Res.

[72] Ares-Santos S, Granado N, Espadas I, Martinez-Murillo R, Moratalla R (2014). Methamphetamine causes degeneration of dopamine cell bodies and terminals of the nigrostriatal pathway evidenced by silver staining. Neuropsychopharmacology.

[73] Groemping Y, Rittinger K (2005). Activation and assembly of the NADPH oxidase: a structural perspective. Biochem J.

[74] Mattson MP (2012). Parkinson’s disease: don’t mess with calcium. J Clin Invest.

[75] Kuwabara T, Imajoh-Ohmi S (2004). LPS-induced apoptosis is dependent upon mitochondrial dysfunction. Apoptosis.

[76] Kitamura O, Takeichi T, Wang EL, Tokunaga I, Ishigami A, Kubo S (2010). Microglial and astrocytic changes in the striatum of methamphetamine abusers. Leg Med.

[77] Kuroda KO, Ornthanalai VG, Kato T, Murphy NP (2010). FosB null mutant mice show enhanced methamphetamine neurotoxicity: potential involvement of FosB in intracellular feedback signaling and astroglial function. Neuropsychopharmacology.

[78] Sekine Y, Ouchi Y, Sugihara G, Takei N, Yoshikawa E, Nakamura K (2008). Methamphetamine causes microglial activation in the brains of human abusers. J Neurosci.

[79] Floyd RA, Carney JM (1992). Free radical damage to protein and DNA: mechanisms involved and relevant observations on brain undergoing oxidative stress. Ann Neurol.

[80] Marí M, Morales A, Colell A, García-Ruiz C, Kaplowitz N, Fernández-Checa JC (2013). Mitochondrial glutathione: features, regulation and role in disease. Biochim Biophys Acta.

[81] Nicholls DG (2009). Mitochondrial calcium function and dysfunction in the central nervous system. Biochim Biophys Acta.

[82] Maragos WF, Jakel R, Chesnut D, Pocernich CB, Butterfield DA, St Clair D (2000). Methamphetamine toxicity is attenuated in mice that overexpress human manganese superoxide dismutase. Brain Res.

[83] Klivenyi P, St Clair D, Wermer M, Yen HC, Oberley T, Yang L (1998). Manganese superoxide dismutase overexpression attenuates MPTP toxicity. Neurobiol Dis.

[84] Zhao Y, Kiningham KK, Lin SM, St Clair DK (2001). Overexpression of MnSOD protects murine fibrosarcoma cells (FSa-II) from apoptosis and promotes a differentiation program upon treatment with 5-azacytidine: involvement of MAPK and NFkappaB pathways. Antioxid Redox Signal.

[85] Yen HC, Oberley TD, Vichibandha S, Ho YS, St Clair DK (1996). The protective role of manganese superoxide dismutase against adriamycin-induced acute cardiac toxicity in transgenic mice. J Clin Invest.

[86] Hald A, Lotharius J (2005). Oxidative stress and inflammation in Parkinson’s disease: is there a causal link ?. Exp Neurol.

[87] Kreutzberg GW (1996). Microglia: a sensor for pathological events in the CNS. Trends Neurosci.

[88] Raineri M, Gonzalez B, Goitia B, Garcia-Rill E, Krasnova IN, Cadet JL (2012). Modafinil abrogates methamphetamine-induced neuroinflammation and apoptotic effects in the mouse striatum. PLoS One.

[89] Zhang W, Wang T, Pei Z, Miller DS, Wu X, Block ML (2005). Aggregated alpha-synuclein activates microglia: a process leading to disease progression in Parkinson’s disease. FASEB J.

[90] Zhang W, Dallas S, Zhang D, Guo JP, Pang H, Wilson B (2007). Microglial PHOX and Mac-1 are essential to the enhanced dopaminergic neurodegeneration elicited by A30P and A53T mutant alpha synuclein. Glia.

[91] Reynolds AD, Kadiu I, Garg SK, Glanzer JG, Nordgren T, Ciborowski P (2008). Nitrated alpha-synuclein and microglial neuroregulatory activities. J Neuroimmune Pharmacol.

[92] Liberatore GT, Jackson-Lewis V, Vukosavic S, Mandir AS, Vila M, McAuliffe WG (1999). Inducible nitric oxide synthase stimulates dopaminergic neurodegeneration in the MPTP model of Parkinson disease. Nat Med.

[93] Rojanathammanee L, Murphy EJ, Combs CK (2011). Expression of mutant alpha-synuclein modulates microglial phenotype in vitro. J Neuroinflammation.

[94] Wu DC, Teismann P, Tieu K, Vila M, Jackson-Lewis V, Ischiropoulos H (2003). NADPH oxidase mediates oxidative stress in the 1-methyl-4-phenyl-1,2,3,6-tetrahydropyridine model of Parkinson’s disease. Proc Natl Acad Sci U S A.

[95] Choi SH, Aid S, Kim HW, Jackson SH, Bosetti F (2012). Inhibition of NADPH oxidase promotes alternative and anti-inflammatory microglial activation during neuroinflammation. J Neurochem.

