# Supplementary material for: Apocynin prevents mitochondrial burdens, microglial activation, and pro-apoptosis induced by a toxic dose of methamphetamine in the striatum of mice via inhibition of p47phox activation by ERK
Source: J Neuroinflammation. 2016 Jan 18;13:12. doi: 10.1186/s12974-016-0478-x (PMC4717833; doi:10.1186/s12974-016-0478-x)
Supplement: Additional file 1: — Supplemental information. Supplemental materials and methods, supplemental results, supplemental references, and supplemental figure legends in detail. (DOCX 41 kb) [file 12974_2016_478_MOESM1_ESM.docx]

**Supplemental information**

**Supplemental Materials and Methods**

**Drug treatment**

To examine whether genetic or pharmacological inhibition of p47phox attenuates methamphetamine (MA)-induced neurotoxicity, two dosing regimens (7 mg/kg, i.p. × 4 or 35 mg/kg, i.p. × 1) of MA were employed. In the multiple dose regimen, WT or p47phox knockout mice received four times of MA (7 mg/kg, i.p.) or saline as a 2-h time interval. In the single administration regimen, WT or p47phox knockout mice received a single dose of MA (35 mg/kg, i.p.) or saline. WT mice were pretreated with apocynin (50 mg/kg, i.p., once a day) for 6 days. On the day of MA injection, additional apocynin was administered at 0.5 h before the 1^st^, 2^nd^, and 4^th^ injection of MA in the multiple dose regimen, and at 0.5 h before the MA injection in the single dose regimen. Mice were sacrificed 1 d and 3 d after the MA injection. The experimental design was shown in Fig. S1.

**Measurement of rectal temperature**

Rectal temperature (under ambient temperature: 21 ± 1 °C) was measured by inserting a thermometer probe lubricated with oil at least 3 cm into the rectum of the mice. To prevent sudden movements, especially in MA-treated mice, animals were gently handled with a wool glove while their tail was moved to allow probe insertion. This was done to reduce any effect of restraint stress on rectal temperature. When the attempt to insert the probe was not successful (i.e., sudden movements of the animal or the need to restrain the mouse), the animal was excluded from the group [1].

**Determination of 4-hydroxynonenal (HNE)**

The amount of lipid peroxidation was determined by measuring the level of 4-hydroxynonenal (HNE) using the OxiSelect^TM^ HNE adduct ELISA kit (Cell Biolabs, Inc., San Diego, CA, U.S.A.) according to the manufacturer’s instructions. 100 μL of cytosolic and mitochondrial fraction at a protein concentration of 10 μg/mL was incubated in the 96-well protein binding plate at 4 °C for overnight. After a protein adsorption, HNE adducts in each well were labeled with HNE antibody, followed by HRP-conjugated secondary antibody. Colorimetric development was then performed with substrate solution. Absorbance was recorded at 450 nm using a microplate reader (Molecular Devices Inc., Sunnyvale, CA, U.S.A.), and an amount of HNE adduct in each sample was calculated from the standard curve of HNE-BSA [2].

**Determination of protein carbonyl**

The extent of protein oxidation was assessed by measuring the content of protein carbonyl groups, which was determined spectrophotometrically with the 2,4-dinitrophenylhydrazine (DNPH)-labeling procedure [2] as described by Oliver et al. [3]. The results are expressed as nmol of DNPH incorporated/mg protein based on the extinction coefficient for aliphatic hydrazones of 21 mM^-1^ cm^-1^. Protein was measured using the Pierce 660 nm Protein Assay^TM^ reagent (Thermo Scientific, Rockford, IL, U.S.A.).

**Supplemental Results**

**Neuroprotection provided by the genetic or pharmacological inhibition of p47phox was more pronounced in the single dose regimen (35 mg/kg × 1) of MA than in the multiple dose regimen (7 mg/kg × 4) of MA in the striatum of mice**

Both multiple (7 mg/kg × 4) and single (35 mg/kg × 1) dose regimens of MA induced significant hyperthermia (both regimens, P < 0.01 vs. Vehicle/WT with saline) in mice. p47phox gene knockout or apocynin significantly attenuated (P < 0.01 vs. Vehicle/WT with MA) hyperthermia induced by a toxic dose of MA (35 mg/kg × 1). However, genetic inhibition of p47phox or apocynin did not significantly affect the hyperthermia induced by repeated MA (7 mg/kg × 4) treatment (Fig. S2).

Similarly, p47phox gene knockout or apocynin significantly attenuated (P < 0.01) decreases in tyrosine hydroxylase-immunoreactivity (TH-IR) and dopamine level, and increase in dopamine turnover rate in the striatum of mice treated with a toxic dose of MA (35 mg/kg × 1), and to a lesser significance degree (P < 0.05) in the mice treated with repeated MA (7 mg/kg × 4) treatment (Figs. S3 and S4).

Since a toxic dose regimen (35 mg/kg × 1) of MA was more sensitive to the neuroprotection provided by the genetic or pharmacological inhibition of p47phox than a multiple dose regimen (7 mg/kg × 4) of MA, we selected a toxic dose of MA (35 mg/kg × 1) for further study.

**ERK inhibitor U0126, apocynin, or p47phox knockout attenuates MA-induced lipid peroxidation and protein oxidation in the striatum of mice; U0126 does not show any additional effect against the protection mediated by apocynin or p47phox knockout**

According to experimental design of Fig. S5, lipid peroxidation and protein oxidation were examined. As shown in Fig S6, the level of 4-hydroxynonenal (HNE) adduct, a marker of lipid peroxidation, was significantly increased in the cytosolic (0.5 h, 1 h, 2 h, 4 h, and 6 h: P < 0.05, P < 0.05, P < 0.01, P < 0.01, and P < 0.05, respectively) or mitochondrial (0.5 h, 1 h, 2 h, 4 h, 6 h, and 1 d: P < 0.01, P < 0.01, P < 0.001, P < 0.001, P < 0.01, and P < 0.05, respectively) fraction of striatum after MA treatment. Increase in HNE level was most prominent 2 h after MA treatment. U0126, apocynin, or p47phox gene knockout significantly attenuated increases in HNE level in the cytosolic (P < 0.05) and mitochondrial (P < 0.01) fractions of striatum 2 h after MA treatment. However, U0126 did not produce additional protection against the attenuation mediated by apocynin or p47phox gene knockout (Fig. S6).

The result obtained from the protein carbonyl measurement, a protein oxidation marker, under our experimental conditions paralleled that obtained from HNE measurement (Fig. S7).

**Morphological characteristics in the microglial activation induced by MA**

In order to assess the activation state of microglia after the MA treatment, skeleton analysis (**a** and **b**) and cell size analysis (**c** and **d**) were performed (Fig. S8). The number of branches (**b-1**), number of junctions (**b-2**), number of triple points (**b-3**), average branch length (**b-4**), and summed branch length (**b-5**) were quantified by skeleton analysis. MA treatment (**a-2**) increased the branching of cell processes and hyper-ramified microglia as compared to saline treatment (**a-1**); MA treatment significantly increased the number of branches (**b-1**), junctions (**b-2**), and triple points (**b-3**). Summed branch length (**b-5**) also significantly increased by MA, but MA did not alter the average branch length (**b-4**). In cell size analysis, average cell size (**d-1**), average cell body size (**d-2**), and cell body size to cell size ratio (%) (**d-3**) were calculated. Representative processed images showed that total cell size (**c-5**) and cell body size (**c-6**) after MA treatment was increased more than total cell size (**c-2**) and cell body size (**c-3**) after saline treatment. Note the unprocessed pictures (**c-1**: saline and **c-2**: MA); MA-induced increases in average cell size (**d-1**), average cell body size (**d-2**), and cell body area to cell area ratio (**d-3**) were observed (Fig. S8).

**Supplemental References**

1. Nguyen XK, Lee J, Shin EJ, Dang DK, Jeong JH, Nguyen TT, Nam Y, Cho HJ, Lee JC, Park DH, Jang CG, Hong JS, Nabeshima T, Kim HC. Liposomal melatonin rescues methamphatemine-elicited mitochondrial burdens, pro-apoptosis, and dopaminergic degeneration through the inhibition PKCδ gene. J Pineal Res. 2015;58(1):86-106.
2. Shin EJ, Shin SW, Nguyen TT, Park DH, Wie MB, Jang CG, Nah SY, Yang BW, Ko SK, Nabeshima T, Kim HC. Ginsenoside Re rescues methamphetamine-induced oxidative damage, mitochondrial dysfunction, microglial activation and dopaminergic degeneration by inhibiting the protein kinase Cδ gene. Mol Neurobiol. 2014;49(3):1400-21.
3. Oliver CN, Ahn BW, Moerman EJ, Goldstein S, Stadtman ER. Age-related changes in oxidized proteins. J Biol Chem. 1987;262:5488-91.

**Supplemental Figure legends**

**Fig. S1** Experimental design I. Effect of inhibition of PHOX on the neurotoxicity induced by a multiple dose regimen (7 mg/kg, i.p. × 4; **a**) or a toxic dose regimen (35 mg/kg, i.p. × 1; **b**) of MA. Apo = apocynin 50 mg/kg, i.p.

**Fig. S2** The effect of inhibition of PHOX on the hyperthermia induced by MA. Effect of apocynin or p47phox knockout on the hyperthermia induced by the multiple doses (7 mg/kg, i.p. × 4; **a**) or a toxic dose (35 mg/kg, i.p. × 1; **b**) of MA. WT = wild-type mice. p47 KO = p47phox knockout mice. Veh = vehicle [10% (v/v) DMSO] for apocynin. Sal = saline. Apo = apocynin (50 mg/kg, i.p.). MA 7 = MA (7 mg/kg, i.p. × 4). MA 35 = MA (35 mg/kg, i.p. × 1). Each value is the mean ± S.E.M. of 12-14 animals. *P < 0.01 vs. corresponding treatment without MA; ^#^P < 0.01 vs. corresponding vehicle/WT with MA (repeated measures ANOVA followed by Fisher’s LSD pairwise comparisons).

**Fig. S3** The effect of inhibition of PHOX on the tyrosine hydroxylase-immunoreactivity (TH-IR) induced by MA. Effect of apocynin or p47phox knockout on the decrease in TH-IR induced by multiple doses (7 mg/kg, i.p. × 4) or a toxic dose (35 mg/kg, i.p. × 1) of MA. WT = wild-type mice. p47 KO = p47phox knockout mice. Veh = vehicle [10% (v/v) DMSO] for apocynin. Sal = saline. Apo = apocynin (50 mg/kg, i.p.). Scale bar = 200 µm. Each value is the mean ± S.E.M. of six animals. *P < 0.01 vs. vehicle/WT with saline; ^#^P < 0.05, ^##^P < 0.01 vs. corresponding vehicle/WT with MA (two-way ANOVA followed by Fisher’s LSD pairwise comparisons).

**Fig. S4** The effect of inhibition of PHOX on the dopaminergic loss induced by MA. Effect of apocynin or p47phox knockout on the changes in dopamine level (**a**) and dopamine turnover rate (**b**) in the striatum induced by the multiple doses (7 mg/kg, i.p. × 4; **a**) or a toxic dose (35 mg/kg, i.p. × 1; **b**) of MA. WT = wild-type mice. p47 KO = p47phox knockout mice. Veh = vehicle [10% (v/v) DMSO] for apocynin. Sal = saline. Apo = apocynin (50 mg/kg, i.p.). Each value is the mean ± S.E.M. of six animals. *P < 0.01 vs. vehicle/WT with saline; ^#^P < 0.05, ^##^P < 0.01 vs. corresponding vehicle/WT with MA (two-way ANOVA followed by Fisher’s LSD pairwise comparisons).

**Fig. S5** Experimental design II. The time-dependent alterations in experimental parameters after MA (35 mg/kg, i.p. × 1) treatment (**a**). Effect of U0126, apocynin, or p47phox knockout on the MA-induced neurotoxicity (**b**). Apo = apocynin (50 mg/kg, i.p.).

**Fig. S6** Effect of U0126, apocynin, or p47phox knockout on the lipid peroxidation after MA.

Cytosolic and mitochondrial changes in the level of 4-hydroxynonenal (HNE) adduct after the MA treatment (**a**), and the effect of U0126, apocynin, or p47phox knockout on the increase in HNE level 2 h after MA (35 mg/kg, i.p.) (**b**). WT = wild-type mice. p47 KO = p47phox knockout mice. Sal = saline. U = U0126 (2 µg, i.c.v.). Apo = apocynin (50 mg/kg, i.p.). V or Veh = vehicle [10% (v/v) DMSO] for U0126 or apocynin. Each value is the mean ± S.E.M. of six animals. *P < 0.05, **P < 0.01, ^&^P < 0.001 vs. saline or vehicle/WT with saline. ^#^P < 0.05, ^##^P < 0.01 vs. vehicle/WT with MA [one-way ANOVA (**a**) or three-way ANOVA (**b**) was followed by Fisher’s LSD pairwise comparisons].

**Fig. S7** Effect of U0126, apocynin, or p47phox knockout on the protein oxidation after MA.

Cytosolic and mitochondrial changes in the level of protein carbonyl after the MA treatment (**a**), and the effect of U0126, apocynin, or p47phox knockout on the increase in protein carbonyl level 2 h after MA (35 mg/kg, i.p.) (**b**). WT = wild-type mice. p47 KO = p47phox knockout mice. Sal = saline. U = U0126 (2 µg, i.c.v.). Apo = apocynin (50 mg/kg, i.p.). V or Veh = vehicle [10% (v/v) DMSO] for U0126 or apocynin. Each value is the mean ± S.E.M. of six animals. *P < 0.05, **P < 0.01, ^&^P < 0.001 vs. saline or vehicle/WT with saline. ^#^P < 0.05, ^##^P < 0.01 vs. vehicle/WT with MA [one-way ANOVA (**a**) or three-way ANOVA (**b**) was followed by Fisher’s LSD pairwise comparisons].

**Fig. S8** Morphological changes in microglia after MA treatment in the striatum. Morphological changes were determined by the analysis of cell skeleton (**a** and **b**) or cell body size (**c** and **d**). Processes were displayed in orange color. Junction points were displayed in purple color and indicated as arrows (**a**). Each value is the mean ± S.E.M. of six animals. *P < 0.05, **P < 0.01, ^&^P < 0.001 vs. saline (one-way ANOVA). Scale bar = 20 (**a**) or 100 (**c**) μm.
