# Supplementary material for: Apocynin prevents mitochondrial burdens, microglial activation, and pro-apoptosis induced by a toxic dose of methamphetamine in the striatum of mice via inhibition of p47phox activation by ERK
Source: J Neuroinflammation. 2016 Jan 18;13:12. doi: 10.1186/s12974-016-0478-x (PMC4717833; doi:10.1186/s12974-016-0478-x)
Supplement: Additional file 2: — Supplemental figures. Fig. S1. Experimental design I. Effect of inhibition of PHOX on the neurotoxicity induced by a multiple dose regimen (7 mg/kg, i.p. × 4; a) or a toxic dose regimen (35 mg/kg, i.p. × 1; b) of MA. Fig. S2. Effect of apocynin or p47phox knockout on the hyperthermia induced by the multiple doses (a) or a toxic dose (b) of MA. Fig. S3. Effect of apocynin or p47phox knockout on the decrease in tyrosine hydroxylase-immunoreactivity induced by multiple doses or a toxic dose of MA. Fig. S4. Effect of apocynin or p47phox knockout on the changes in dopamine level (a) and dopamine turnover rate (b) in the striatum induced by the multiple doses or a toxic dose of MA.Fig. S5. Experimental design II. The time-dependent alterations in experimental parameters after MA (35 mg/kg, i.p. × 1) treatment (a). Effect of U0126, apocynin, or p47phox knockout on the MA-induced neurotoxicity (b). Fig. S6. Cytosolic and mitochondrial changes in the level of 4-hydroxynonenal (HNE) adduct after the MA treatment (a), and the effect of U0126, apocynin, or p47phox knockout on the increase in HNE level 2 h after MA (35 mg/kg, i.p. × 1) (b).Fig. S7. Cytosolic and mitochondrial changes in the level of protein carbonyl after the MA treatment (a), and the effect of U0126, apocynin, or p47phox knockout on the increase in protein carbonyl level 2 h after MA (35 mg/kg, i.p. × 1) (b). Fig. S8. Morphological changes in microglia after MA treatment in the striatum. Morphological changes were determined by the analysis of cell skeleton (a, b) or cell body size (c, d). Detailed figure legends are included in the Additional file 1. (PDF 14030 kb) [file 12974_2016_478_MOESM2_ESM.pdf]

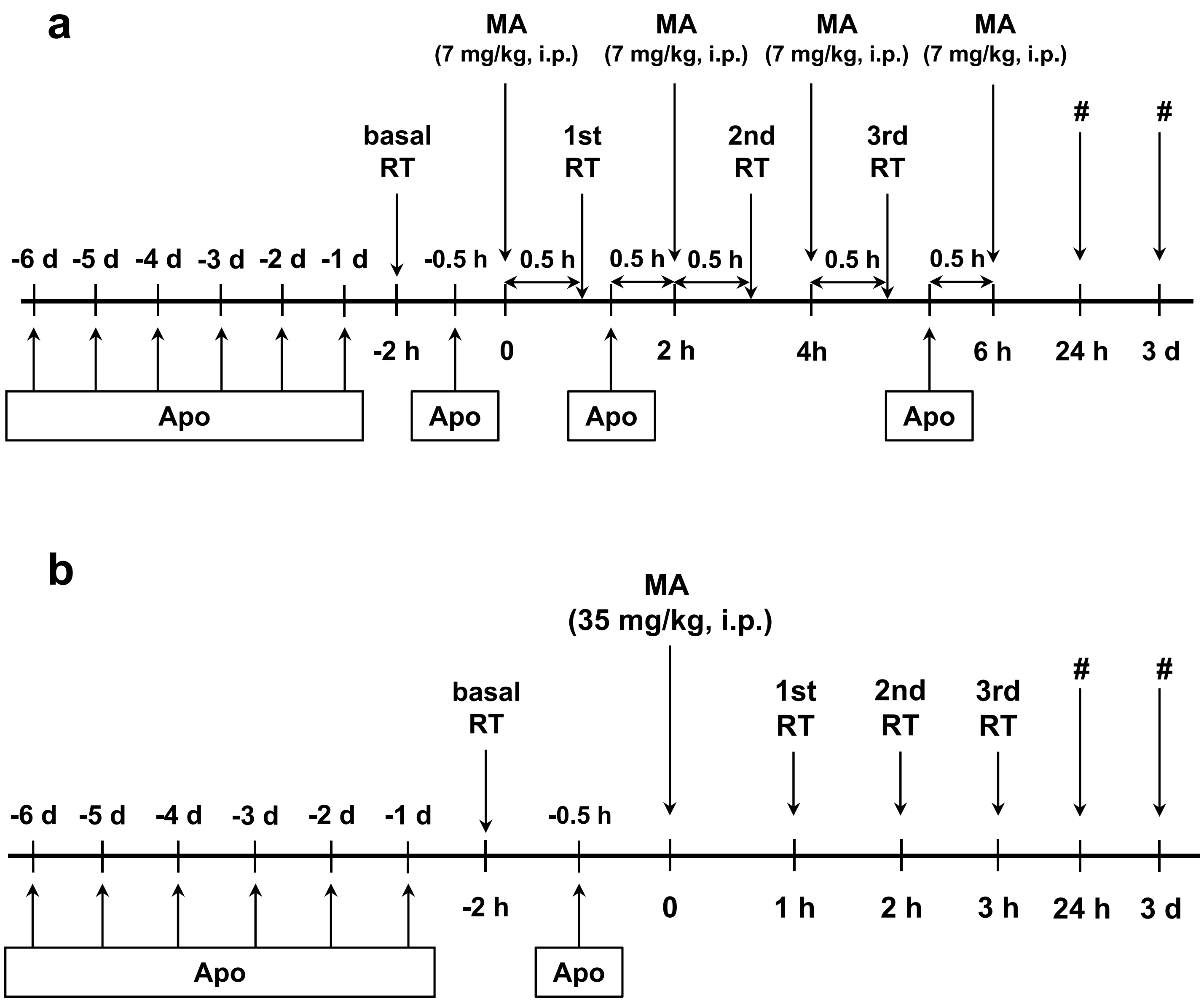

RT: Rectal temperature

#: Sacrifice

Fig. S1

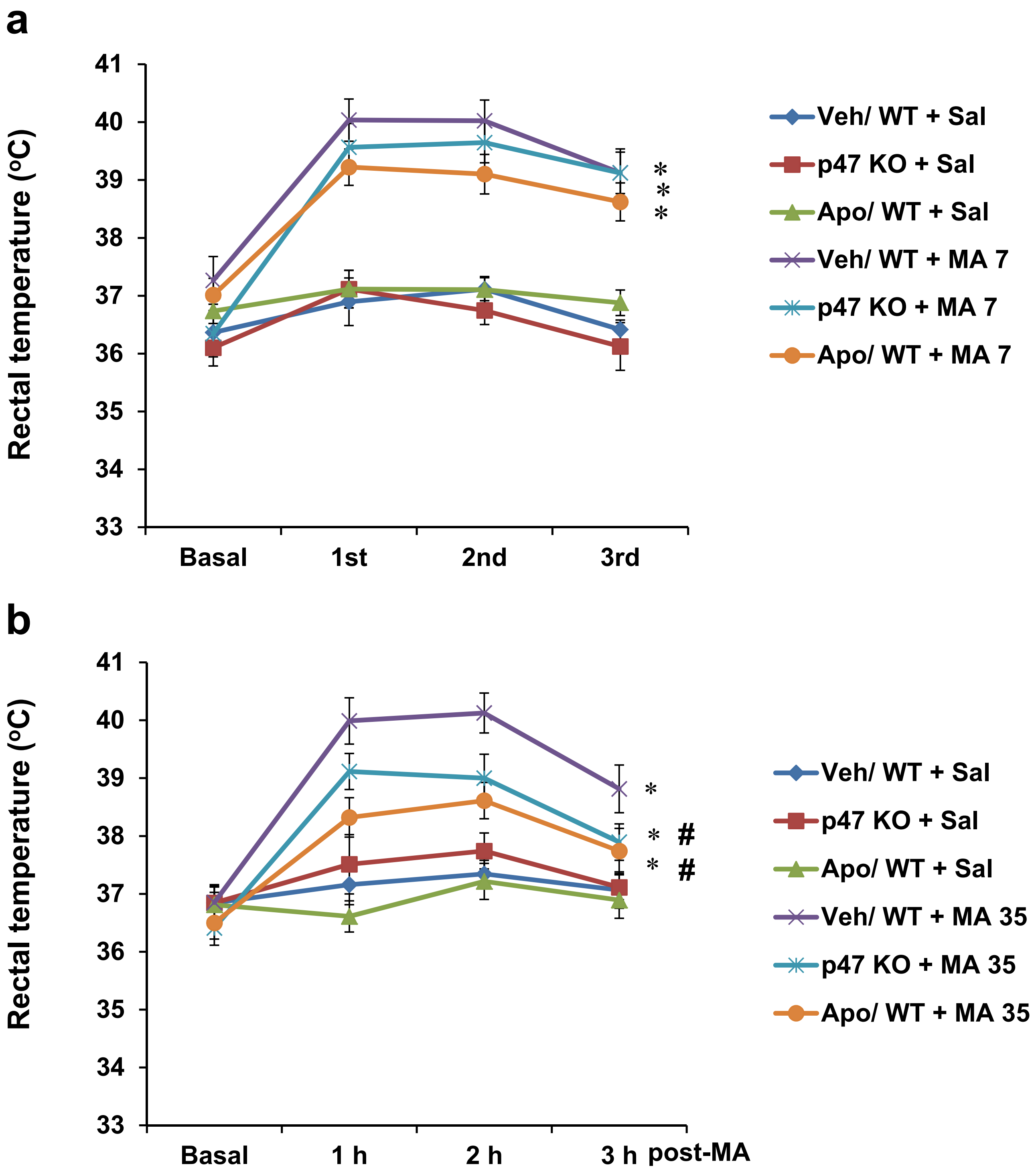
a
Rectal temperature (oC)
41
40
39
38
37
36
35
34
33
Basal
1st
2nd
3rd
*
*
*
Veh/ WT + Sal
p47 KO + Sal
Apo/ WT + Sal
Veh/ WT + MA 7
p47 KO + MA 7
Apo/ WT + MA 7
b
Rectal temperature (oC)
41
40
39
38
37
36
35
34
33
Basal
1 h
2 h
3 h post-MA
*
* #
* #
Veh/ WT + Sal
p47 KO + Sal
Apo/ WT + Sal
Veh/ WT + MA 35
p47 KO + MA 35
Apo/ WT + MA 35

Fig. S2

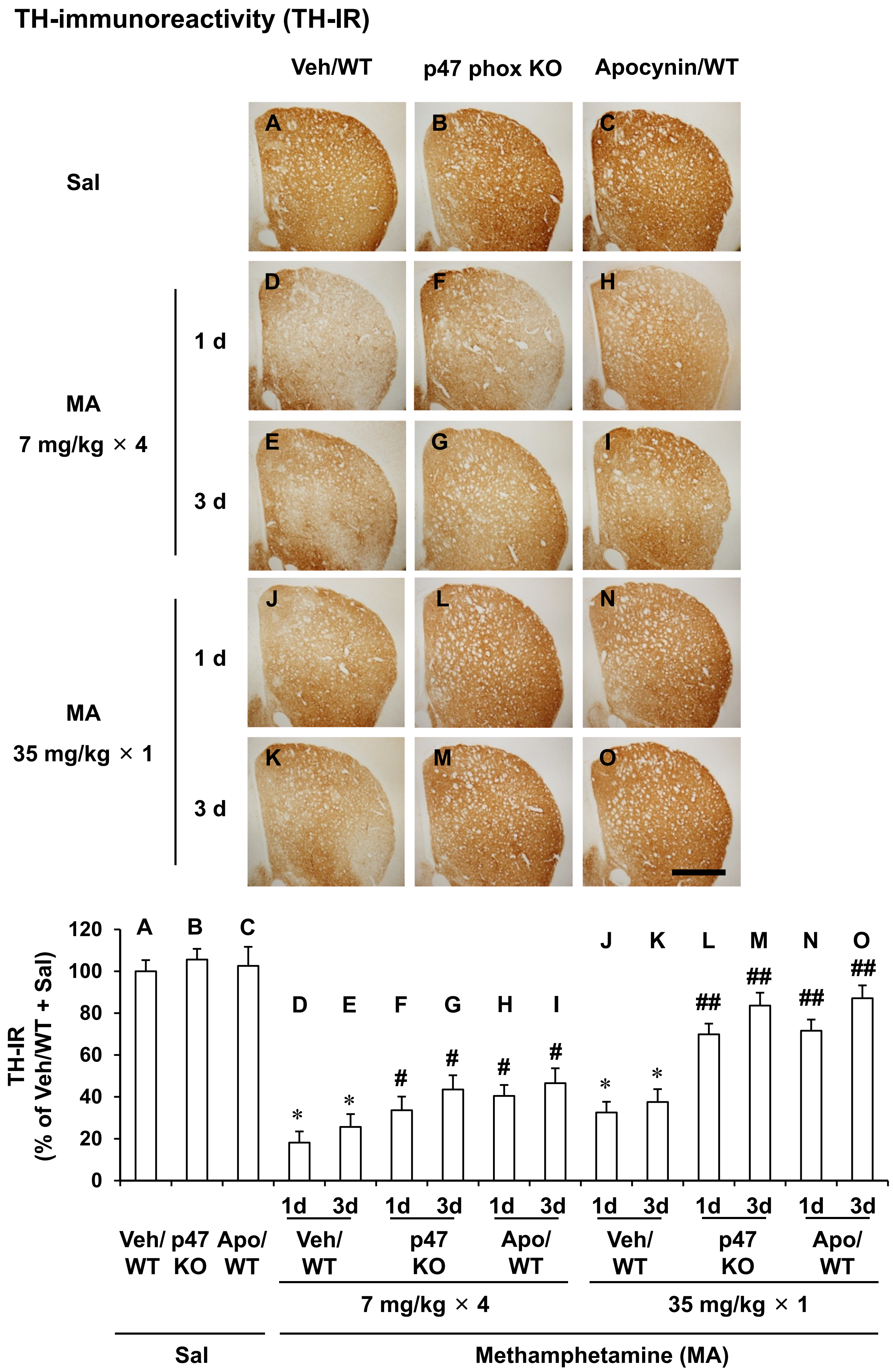
TH-immunoreactivity (TH-IR)
Veh/WT
p47 phox KO
Apocynin/WT
Sal
MA
7 mg/kg × 4
1 d
3 d
MA
35 mg/kg × 1
1 d
3 d
A
B
C
D
E
F
G
H
I
J
K
L
M
N
O
TH-IR
(% of Veh/WT + Sal)
0
20
40
60
80
100
120
*
#
##
1d
3d
Veh/ WT
p47 KO
Apo/ WT
7 mg/kg × 4
35 mg/kg × 1
Sal
Methamphetamine (MA)

Fig. S3

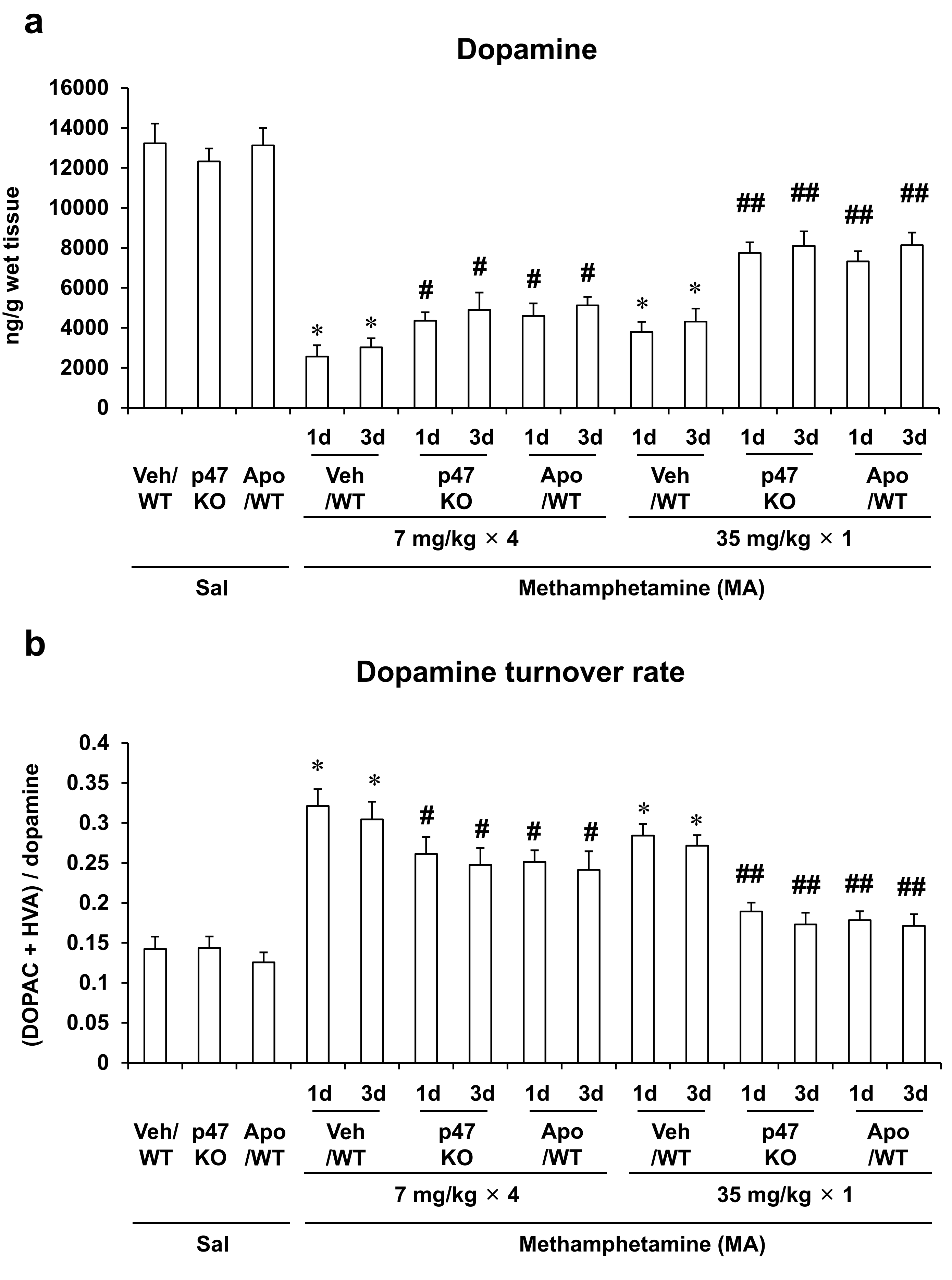
a
Dopamine
ng/g wet tissue
16000
14000
12000
10000
8000
6000
4000
2000
0
*
*
#
#
#
#
*
*
##
##
##
##
1d 3d 1d 3d 1d 3d 1d 3d 1d 3d 1d 3d
Veh/ WT
p47 KO
Apo /WT
Veh /WT
p47 KO
Apo /WT
Veh /WT
p47 KO
Apo /WT
7 mg/kg × 4
35 mg/kg × 1
Sal
Methamphetamine (MA)
b
Dopamine turnover rate
(DOPAC + HVA) / dopamine
0.4
0.35
0.3
0.25
0.2
0.15
0.1
0.05
0
*
*
#
#
#
#
*
*
##
##
##
##
1d 3d 1d 3d 1d 3d 1d 3d 1d 3d 1d 3d
Veh/ WT
p47 KO
Apo /WT
Veh /WT
p47 KO
Apo /WT
Veh /WT
p47 KO
Apo /WT
7 mg/kg × 4
35 mg/kg × 1
Sal
Methamphetamine (MA)

Fig. S4

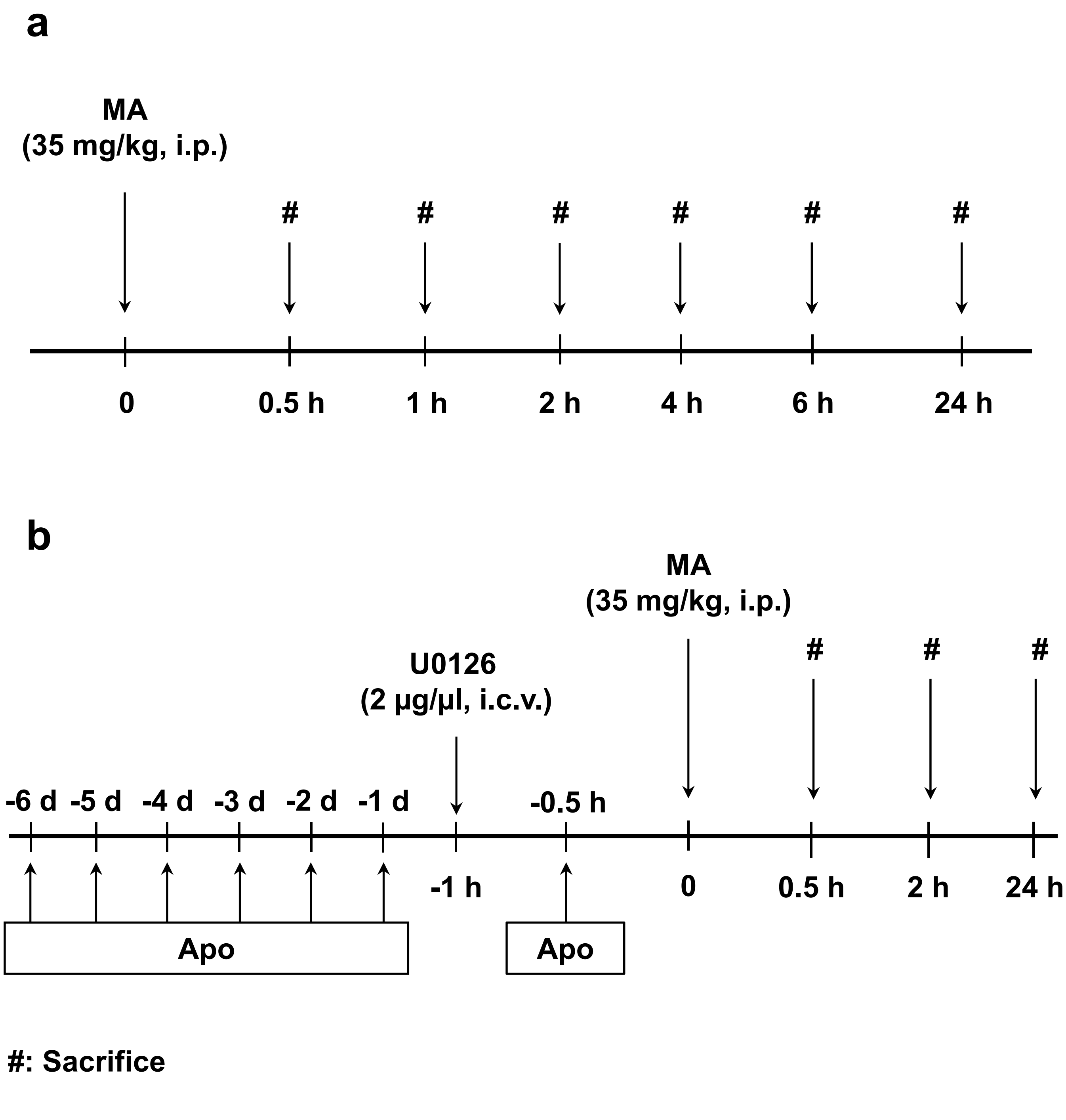
a
MA
(35 mg/kg, i.p.)
#
0
0.5 h
1 h
2 h
4 h
6 h
24 h
b
MA
(35 mg/kg, i.p.)
U0126
(2 µg/µl, i.c.v.)
-6 d
-5 d
-4 d
-3 d
-2 d
-1 d
-0.5 h
-1 h
0
0.5 h
2 h
24 h
Apo
Apo
#: Sacrifice

Fig. S5

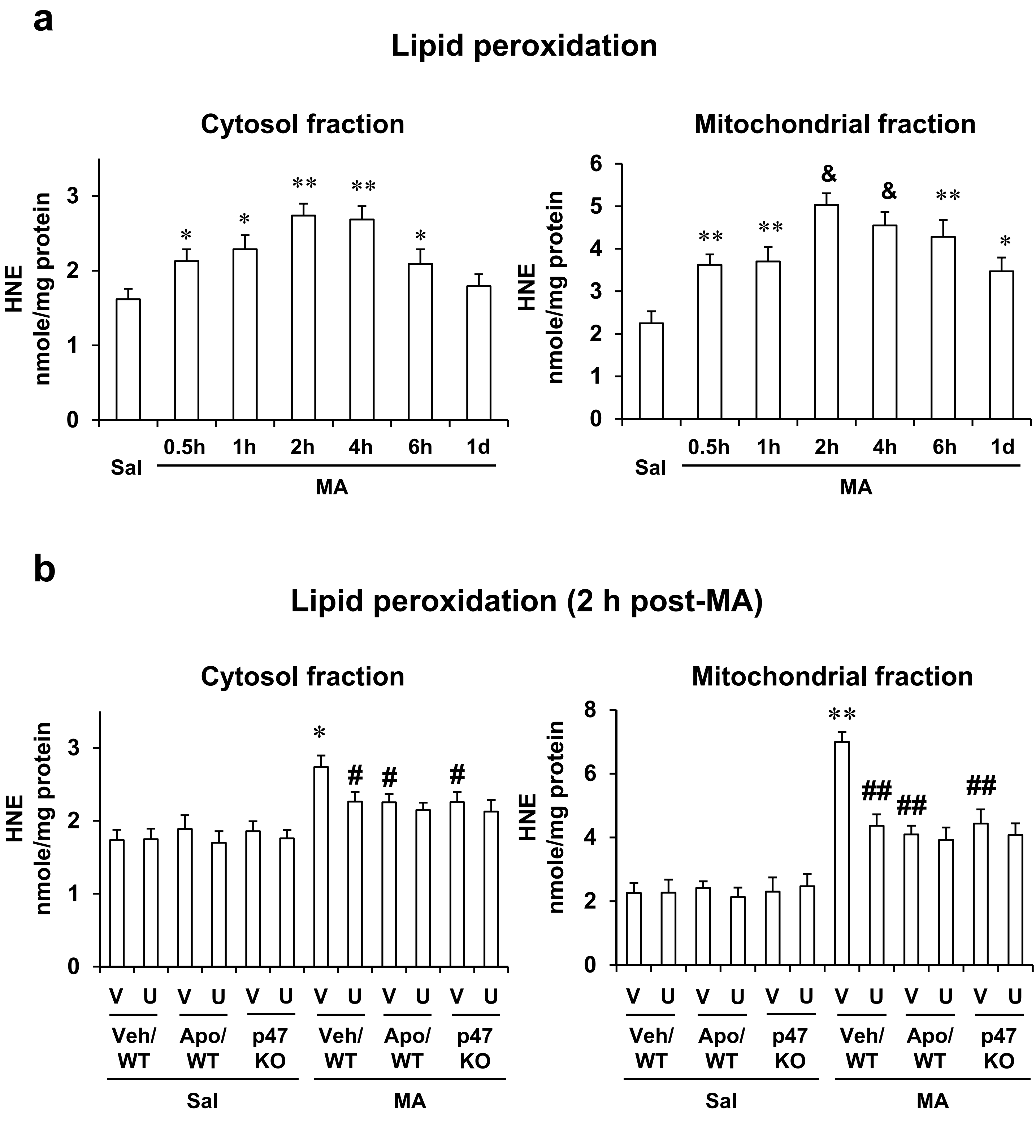
a
Lipid peroxidation
Cytosol fraction
HNE
nmole/mg protein
0
1
2
3
*
*
**
**
*
Sal
0.5h
1h
2h
4h
6h
1d
MA
Mitochondrial fraction
HNE
nmole/mg protein
0
1
2
3
4
5
6
**
**
&
&
**
*
Sal
0.5h
1h
2h
4h
6h
1d
MA
b
Lipid peroxidation (2 h post-MA)
Cytosol fraction
HNE
nmole/mg protein
0
1
2
3
*
#
#
#
V U V U V U V U V U V U
Veh/ WT
Apo/ WT
p47 KO
Veh/ WT
Apo/ WT
p47 KO
Sal
MA
Mitochondrial fraction
HNE
nmole/mg protein
0
2
4
6
8
**
##
##
##
V U V U V U V U V U V U
Veh/ WT
Apo/ WT
p47 KO
Veh/ WT
Apo/ WT
p47 KO
Sal
MA

Fig. S6

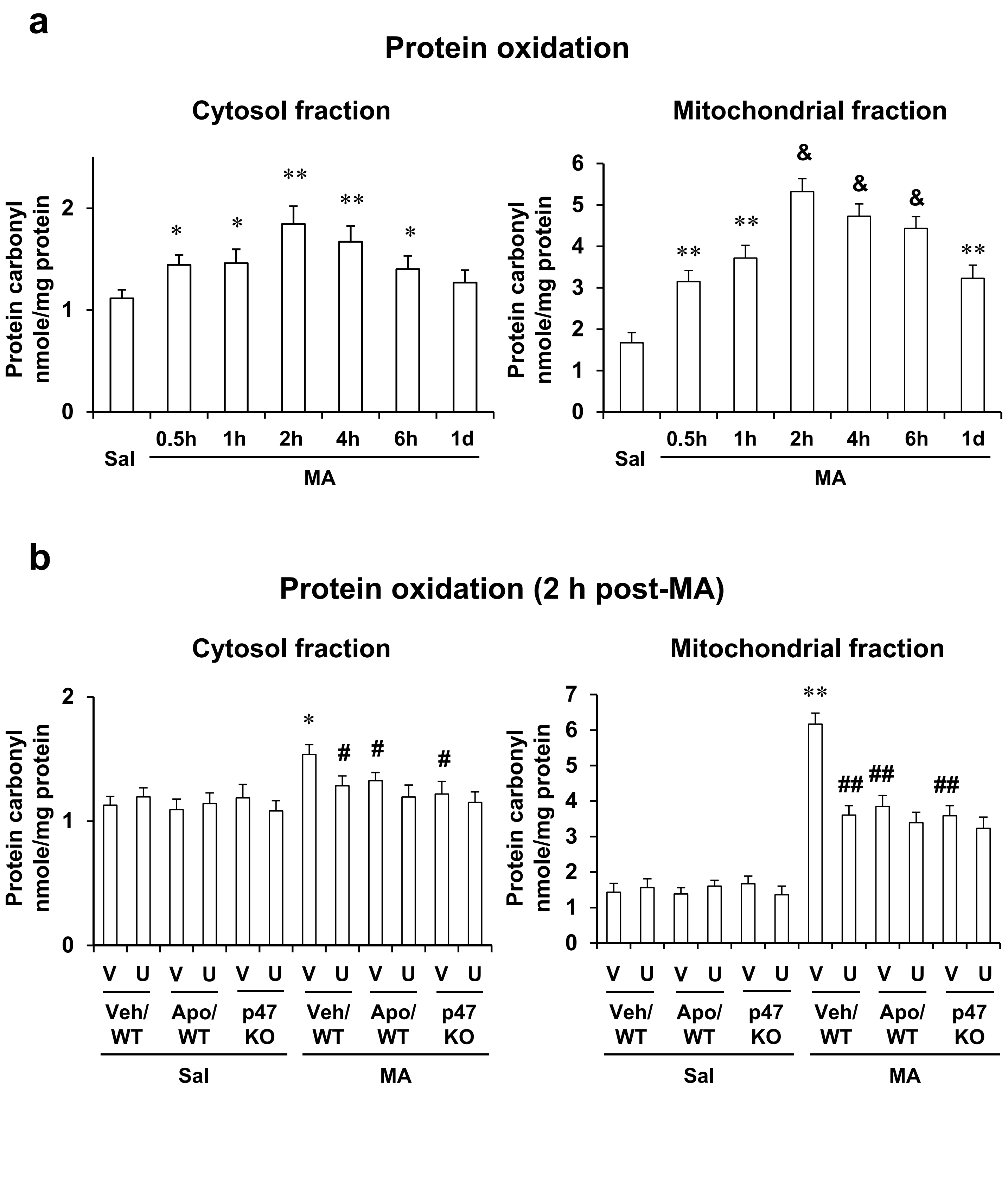
a
Protein oxidation
Cytosol fraction
Mitochondrial fraction
Protein carbonyl nmole/mg protein
Sal
0.5h
1h
2h
4h
6h
1d
MA
b
Protein oxidation (2 h post-MA)
Cytosol fraction
Mitochondrial fraction
Protein carbonyl nmole/mg protein
V U
Veh/ WT
Apo/ WT
p47 KO
Sal
MA

Fig. S7

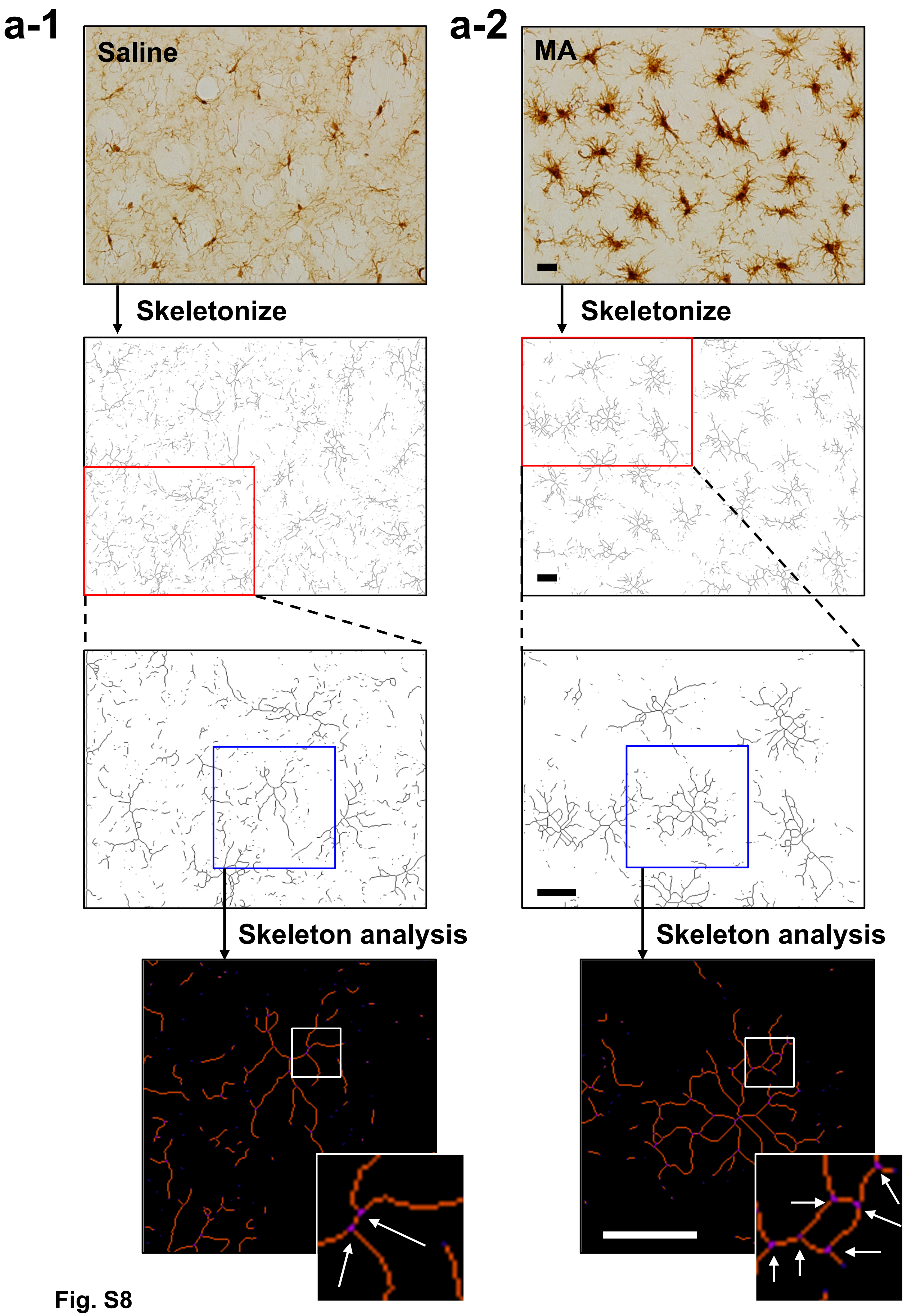
a-1
Saline
Skeletonize
Skeleton analysis
a-2
MA
Skeletonize
Skeleton analysis

Fig. S8

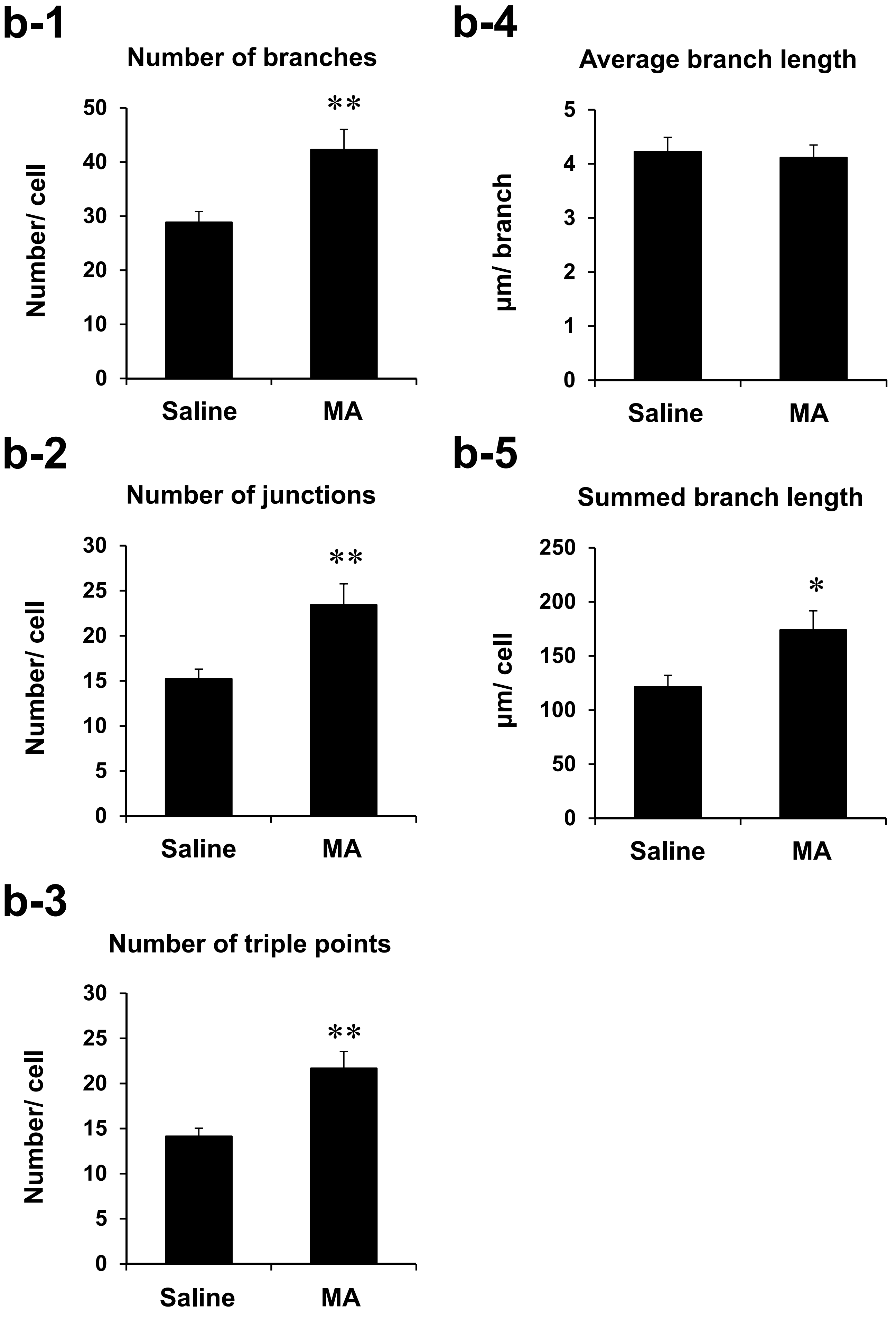

Fig. S8 (continued)

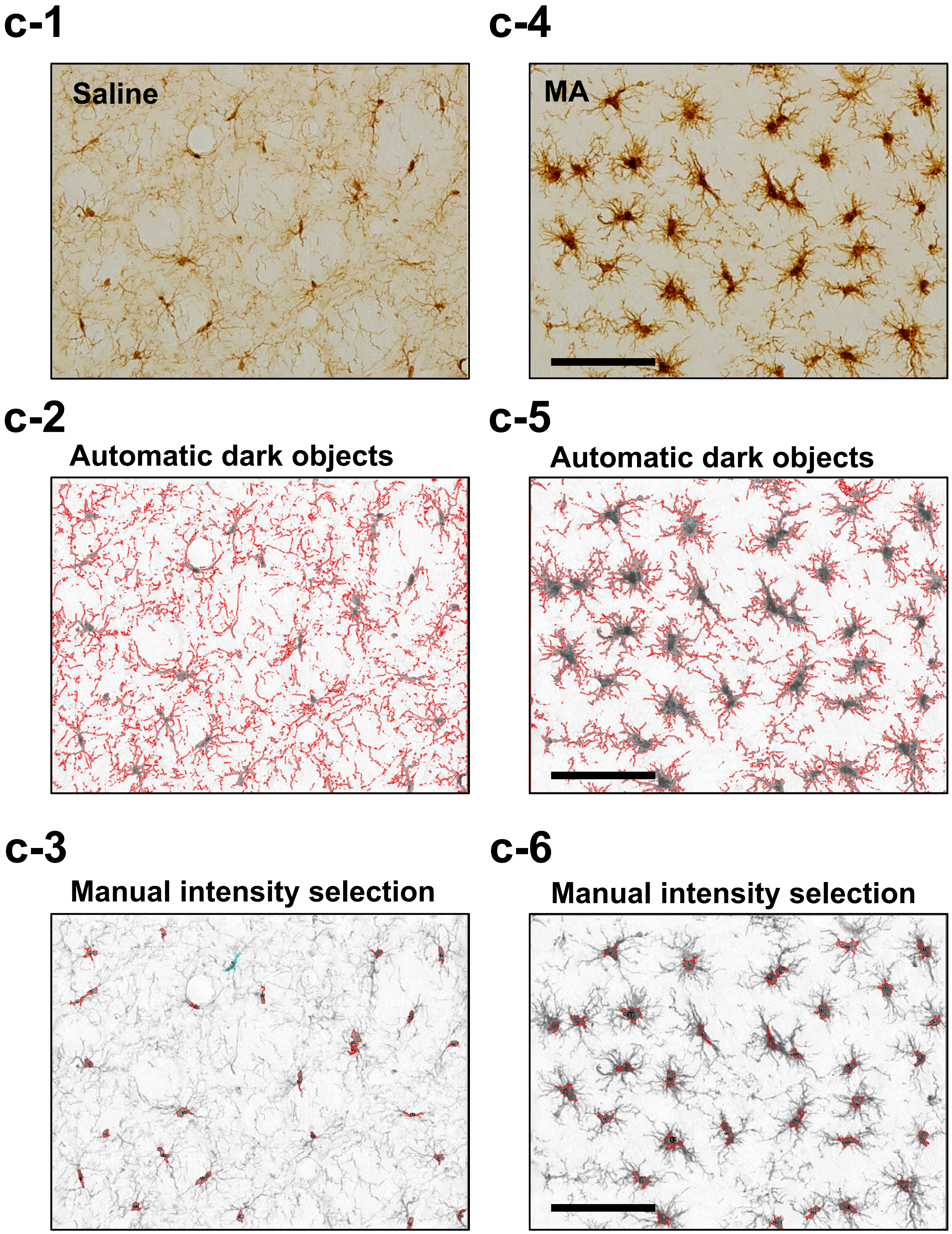

Fig. S8 (continued)

d-1

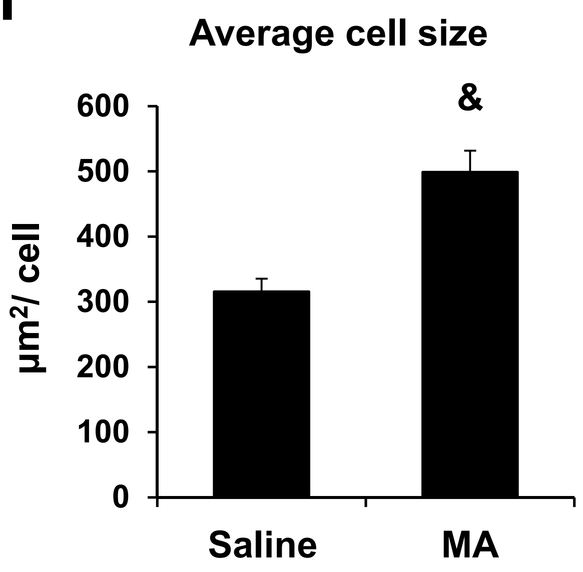

d-2

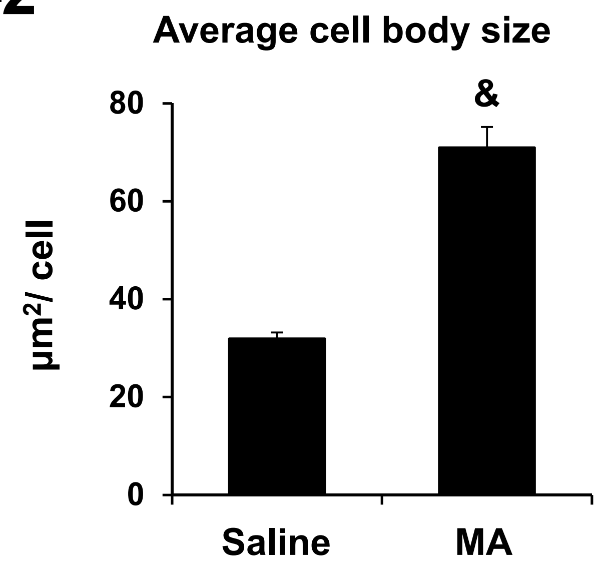

d-3

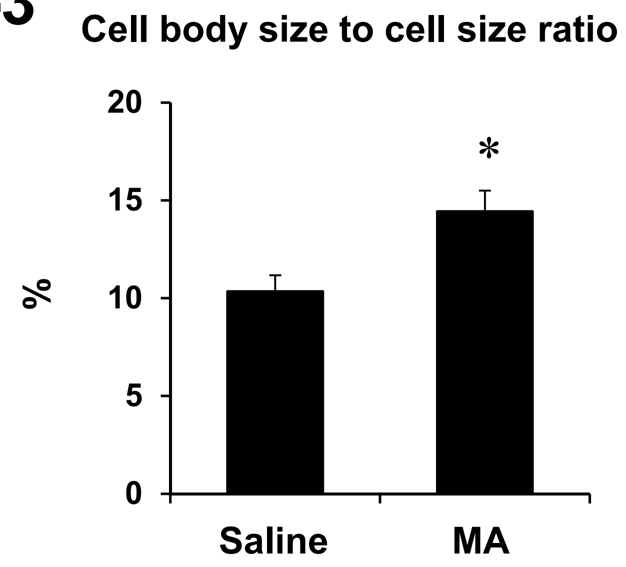

Fig. S8 (continued)
